# ‘Just snap out of it’ – the experience of loneliness in women with perinatal depression: a Meta-synthesis of qualitative studies

**DOI:** 10.1186/s12888-023-04532-2

**Published:** 2023-02-28

**Authors:** Katherine Adlington, Cristina Vasquez, Eiluned Pearce, Claire A. Wilson, Rebecca Nowland, Billie Lever Taylor, Sarah Spring, Sonia Johnson

**Affiliations:** 1 Division of Psychiatry, University College London, London, UK; 2 Section of Women’s Mental Health, King’s College London, London, UK; 3 East London NHS Foundation Trust, London, UK; 4 South London and Maudsley NHS Foundation Trust, London, UK; 5 School of Health and Midwifery, University of Central Lancashire, Preston, UK; 6 Person with lived experience, London, UK; 7 Camden and Islington NHS Foundation Trust, London, UK

**Keywords:** Perinatal depression, Postpartum depression, Postnatal depression, Antenatal depression, Loneliness, Qualitative metasynthesis, Qualitative synthesis

## Abstract

**Background:**

Pregnancy and the arrival of a new baby is a time of great transition and upheaval. Women often experience social isolation and loneliness at this time and may develop depression, particularly in the postnatal period. Qualitative studies have reported that loneliness is also a feature of perinatal depression. However, until now there has been no attempt to synthesise research exploring the links between loneliness and perinatal depression. This study’s aim was to explore existing qualitative evidence to answer two research questions: What are the experiences of loneliness for women with perinatal depression? What helps and what makes loneliness worse for women with perinatal depression?

**Methods:**

A qualitative meta-synthesis retrieved primary qualitative studies relevant to the research questions. Four electronic databases were systematically searched (Ovid MEDLINE®; PsycINFO; Embase; Web of Science). Papers were screened according to pre-defined inclusion criteria and assigned a quality score. Thematic analysis was used to identify major overarching themes in the literature.

**Results:**

Twenty-seven relevant qualitative studies were included. Themes relating to the interaction between perinatal depression and loneliness included self-isolation and hiding symptoms due to stigma of perinatal depression and fear of judgement as a ‘bad mother’; a sudden sense of emotional disconnection after birth; and a mismatch between expected and actual support provided by partner, family and community. There was also a double burden of loneliness for women from disadvantaged communities, due to increased stigma and decreased social support. Validation and understanding from healthcare professionals, peer support from other mothers with experience of perinatal depression, and practical and emotional family support were all important factors that could ameliorate loneliness.

**Conclusions:**

Loneliness appears to play a central role in the experience of perinatal depression based on the frequency with which it emerged in women’s accounts. The findings provide a foundation for the development of further theories about the role of loneliness in perinatal depression and evidence in which future psychological and social intervention design processes can be rooted. Addressing stigma and offering culturally appropriate professional and peer support are potential targets for interventions that could help women with perinatal depression, particularly in disadvantaged communities, feel less lonely.

**Trial registration:**

Prospero registration: https://www.crd.york.ac.uk/prospero/display_record.php? RecordID = 251,936.

**Supplementary Information:**

The online version contains supplementary material available at 10.1186/s12888-023-04532-2.

## Background

The transition to motherhood – including pregnancy and the arrival of a new baby – is a period of enormous change in a woman’s life, with significant transformations in their intimate relationships, social networks, careers, values and routines [1, 2]. Despite common societal expectations that it should be the ‘happiest time of their life’, women are vulnerable to developing psychological distress and mental health problems during the perinatal period. In particular, depression affects approximately one in six pregnant women [3] and one in five women during the first 3 months after birth [4].

It is important to recognise and treat perinatal depression because it significantly affects women’s quality of life and relationships [5]. It increases their risk of self-harm or death by suicide [6]. It also impairs mother-infant interactions and can have long-term adverse effects on a child’s cognitive and emotional development [7, 8]. Understanding factors, especially remediable ones, that place new mothers at increased risk of depression is therefore important. One such factor is the potential role of loneliness and lack of social support in contributing to and maintaining the onset of perinatal depression. Loneliness is defined as a negative emotional state that arises when there is a perceived discrepancy between desired and actual social relationships [9]. Loneliness has been conceptualised as having three key aspects: ‘emotional loneliness’ (the absence of close attachment with another significant person or persons); ‘social loneliness’ (the lack of integration into social networks) and ‘existential loneliness’ (stemming from the realisation that a human being is fundamentally alone) [10, 11].

Women are vulnerable to loneliness during their transition to motherhood [12, 13]. A recent poll in the UK found more than half of parents had experienced loneliness since becoming a parent and a fifth had felt lonely in the last week [14]. Another small UK poll found that 32% of mothers aged under 25 with a young child felt lonely always or often [15]. Similarly, a longitudinal study in Finland found that 38% of pregnant women felt lonely at least some of the time [16]. Recent cross-national online research emerging from the Covid-19 pandemic has found that 53.5% of perinatal women have experienced high levels of loneliness during the pandemic [17]. Qualitative evidence shows new mothers link their loneliness to a lack of social contact, a change in their sense of identity, a lack of empathy and support from peers and family and critical self-comparisons with perceived mothering norms [12, 18].

Loneliness has been shown to have substantial effects on mental and physical health and the relationships tend to be bidirectional [19]. Most research exploring the interaction between loneliness and mental health has focused on depression. Cross-sectional studies show that people with depression are 10 times more likely to feel lonely than the general population [20]. Longitudinal studies have shown that loneliness increases the risk of depression [21] and worsens depressive symptoms in those already depressed [22]. The mechanisms behind this complex, reciprocal inter-relationship between loneliness and depression are unclear [23]. Theoretical models propose that loneliness has biological, social and cognitive consequences that can increase the risk of depression [24].

There is increasing evidence that loneliness is also a risk factor for depression in the perinatal period [25, 26]. However, the unique circumstances associated with the transition to motherhood, and the increased risk of depression at this time, suggests that loneliness may have a unique interaction with perinatal depression. Small qualitative studies that have been conducted exploring the more general experience of social support for women with perinatal depression have found that loneliness is raised as an important experience, with women describing themselves as “enveloped in unbearable loneliness” [27], “detached and removed from those around them” [28] and, experiencing “a deep sense of loneliness” [29]. To our knowledge only one qualitative study has specifically focused on loneliness in perinatal depression and found women repeatedly described feeling lost, alone and isolated and linked these feelings to lack of supportive relationships, fears of judgement and dislocation from their previous lives and identities [18]. Many studies focus on the social experiences of more marginalised groups with perinatal depression (such as teenage, ethnic minority and immigrant mothers) and find that loneliness is complicated by the multiple disadvantages that these women face [29–31].

However, to date there has been no attempt to systematically search and synthesise these existing small-scale and disparate qualitative studies that explore both perinatal depression and loneliness. Using a qualitative meta-synthesis methodology, we aimed to provide an overview of the available international research, to gain a higher-order understanding of the data and to identify major overarching themes about the role loneliness plays in the day-to-day lives of women with perinatal depression. Intersectionality theory states that factors like gender can interact with other socioeconomic categories, such as class and race, to influence the exclusion and marginalisation of people in society [32–34]. Loneliness and perinatal depression may not be experienced equally by all women [35], thus an intersectional lens was used in this analysis to consider the experience of different cohorts of women in different sociocultural settings and contexts.

The resulting synthesised qualitative evidence also has the potential to enhance understanding of the support women with perinatal depression need to feel less lonely and inform the development of social and psychological interventions that specifically target loneliness with a potential dual aim of also reducing the risk of perinatal depression.

## Methods

A meta-synthesis of qualitative studies was conducted exploring loneliness experiences among women with perinatal depression to answer the following two research questions: What are the experiences of loneliness for women with perinatal depression? What helps and what makes loneliness worse for women with perinatal depression?

The ENTREQ statement was used to guide the design, conduct and reporting of this qualitative meta-synthesis [36].

### Design

This qualitative meta-synthesis was conducted in keeping with the processes and 6 step approach defined by Lachal et al [37, 38]. Qualitative meta-synthesis is a relatively new rigorous research technique – adapted from thematic synthesis methodology [39] - that involves systematically searching for qualitative literature using a systematic review protocol followed by the synthesis of results from included primary qualitative research studies to identify common themes. A meta-synthesis provides a useful summary of the wider literature, a broader understanding of a topic and generates a higher-order synthesis, potentially resulting in new overarching themes [37, 40–42].

### Database and search strategy

The pre-planned meta-synthesis protocol and search strategy was registered on Prospero (https://www.crd.york.ac.uk/prospero/display_record.php? RecordID = 251,936). The search was conducted using the following four electronic bibliographical databases, from inception to 26th July 2021: Ovid MEDLINE® (1946-present); PsycINFO (1806-present); Embase (1947 – present); and Web of Science (1900-present). Forward and backward citation tracking was performed on eligible articles.

Search terms captured the subjective experience of loneliness in women with perinatal depression using a qualitative approach. Search terms captured the 4 key concepts: (i) a perinatal population, (ii) mental health disorders, (iii) loneliness and (iv) qualitative research. Additional file 1 (see Supplementary Materials) describes the search strategy and search terms used in full, including both free text and MeSH terms used (Additional file 1: Table 1).

The search strategy was designed to be inclusive - using search terms that were related and conceptually similar to loneliness, such as social isolation, social network, social support and social connection [22, 43–45]. These conceptually overlapping terms were included in this search to ensure comprehensive retrieval of relevant papers [9, 46]. On direct analysis of the results sections, only primary data that appeared directly related to the experience of loneliness were extracted and included in the synthesis.

The initial search included search terms for all perinatal mental health disorders resulting in a large number of papers, therefore, when screening using inclusion and exclusion criteria, a pragmatic decision was made to only include papers concerned with perinatal depression, resulting in a more manageable number of papers and a more focused review. To increase the comprehensiveness of our search, studies were included if a majority of the women in the study (> 50%) met formal diagnostic criteria for depression, or received high risk scores on depression scales or self-reported a diagnosis of perinatal depression. To enhance the clinical relevance of the review, an extended definition of the perinatal period - up to 2 years postnatal - was used in keeping with recent changes in the UK extending National Health Service perinatal mental health provision up to 2 years after birth [47].

STARLITE principles were used to report on the overall literature search [37].

### Selection: Inclusion and exclusion criteria:

Studies were screened in accordance with the inclusion and exclusion criteria listed in Table 1.

**Table 1:** Inclusion and Exclusion Criteria

	Inclusion	Exclusion
**Participants**	● > 50% study participants had personally experienced depression in the perinatal period, whether new episode or continuing episode of pre-existing diagnosis ● Discussing current episode or recalling details retrospectively ● Perinatal depression either diagnosed by clinicians, detected through screening questionnaires (such as the Edinburgh Postnatal Depression scale) or self-identified ● Participants who had experienced a pregnancy of any duration and a live birth or a miscarriage or a stillbirth. ● Perinatal period defined as pregnancy up to 2 years post birth ● No age restrictions ● No restrictions on other physical or mental health co-morbidities (apart from substance misuse)	● Participants with a co-morbid diagnosis of substance misuse disorder. ● Participants who have experienced distress, bereavement, loss, grief or trauma during the perinatal period but without a diagnosis of perinatal depression ● Studies including qualitative data collected from health care professionals, partners or other people with close experience of and interactions with women with perinatal depression. ● Participants discussing experience of depression outside the perinatal period
**Concept**	● Any primary research study with a qualitative research design to explore a participant’s experience, including any data collection method (such as interviews, focus groups, diaries or online data collection) and any method of qualitative analysis of primary data (such as grounded theory, ethnography, thematic analysis, interpretive phenomenological analysis (IPA), framework approach, or narrative analysis). ● Majority (> 50%) of the results section concerned with participants discussing their subjective experiences of loneliness or closely-related themes (such as perceived social isolation, lack of connection or lack of social support) associated with their perinatal depression.	● Existing qualitative meta-syntheses or reviews ● Mixed methods studies (the qualitative element of these studies did not tend to be of high enough quality). ● Case studies or ethnographic exploration with only one participant ● Conference abstracts, PhD theses, dissertations or other types of grey literature. ● Studies evaluating an intervention.
**Context**	● Studies in any geographical or cultural setting.	
**Language**	● English only	● Non-English

### Data screening

Titles and abstracts were screened, followed by full text screening by two independent reviewers (KA screening 100% and CV screening 10%). Results were discussed to check for agreement and disagreements were discussed for adjudication with the wider review team at regular meetings. There was a high level of agreement between both researchers performing screening, extraction and quality appraisal throughout the process.

### Quality appraisal

The Critical Appraisal Skills Programme (CASP) qualitative research checklist was used by two independent researchers to assign each paper a quality score (KA 100% and CV 10% of papers) (see Table 3) [48, 49]. There was 93% agreement on the quality scores of the 10% of papers that both researchers scored and agreement was reached on all papers after a further discussion between both researchers. Articles were not excluded on the basis of low quality as the aim of the review was to produce a comprehensive synthesis based on all relevant data. Instead, the quality scores were considered when formulating the synthesis and deciding which data to prioritise, as suggested in methodological guidance [40, 41].

### Data extraction and analysis

The method of data analysis was a meta-synthesis using citations to evidence the thematic analysis and resulting framework.

Study characteristics (such as sample size, diagnosis, data collection and analysis methods) were recorded by two independent reviewers (KA 100% and CV 10% of papers) using a proforma, and are summarised in Table 2, with 100% agreement. In terms of extraction of primary data, one researcher (KA) immersed herself in the data by carefully reading and re-reading all eligible papers. Any text found in the Results section of eligible papers relevant to the meta-synthesis aims (either direct quotes or analysis of primary data) was identified and highlighted using a qualitative data analysis software package, Nvivo 20 [50]. A second researcher (CV) independently reviewed a randomly selected 10% of eligible articles results sections to confirm agreement with the first researcher’s selection of relevant text.

**Table 2:** Characteristics of included studies

Author & ref	Year	Participants			Study setting		Data collection & analysis		Quality assessment (score out of 10)
		Sample size	Diagnosis		Country	Clinical setting recruited	Data collection	Data analysis	
Beck [27]	1992	7	Postpartum depression	Self report	USA	Postpartum depression support group	One-to-one interviews	Colaizzi’s phenomenological 6-step data analysis method	6
Blanchard [51]	2009	7	Antenatal depresssion	EPDS> = 10	USA	Family health centre - primary care & counselling	Semi-structured interviews	Colaizzi’s phenomenological data analysis method	9
Boath [52]	2013	15	Postpartum depression	EPDS> 12	England	Health visitors in the community	Semi-structured interviews	Thematic framework analysis	9
Edhborg [53]**	2005	22	Postpartum depression	EPDS> = 10	Sweden	Maternity ward	Unstructured interviews	Grounded theory	9
Evans [54]	2012	512 online postings (unknown number of participants)	Postpartum depression	Self report	Study based in Canada (unknown location of participants)	Online postpartum depression support group	Unit of analysis was individual online messages	Directed content analysis	7
Gardner [55]	2014	6	Postpartum depression	EPDS> = 10	England	NHS commissioned parenting groups.	Semi-structured interviews	Interpretative Phenomenological Analysis	8
Hanley [56]	2006	10	Postnatal depression	EPDS> = 12 & clinical diagnosis	Wales	General Practice/ primary care.	Semi-structured interviews	Content analysis using Colaizzi’s method	6
Highet [28]	2014	28	Postnatal depression	Self report	Australia	Website of 3rd sector organisation	24 Face to face and 4 telephone interviews	Grounded theory	8
Jackson [57]	2020	21	Antenatal or postnatal depression.	Self report	England	Flyers, health visitors, midwives, support groups and local press.	Semi-structured interviews (18 face-to-face & 3 telephone)	Thematic analysis	10
Keefe [58]	2016	30	Postpartum depression	Self report	USA	Flyers in an urban health centre serving low-income residents.	Semi-structured face-to-face interviews	Constant comparative analysis	4
Keefe [59]	2019	30	Postpartum depression	Self report	USA	Flyers in an urban health centre serving low-income residents	Semi-structured face-to-face interviews	Constant comparative analysis	5
Letourneau [60]	2007	41	Postpartum depression	Self report	Canada	Newspapers and community health care clinics	41 Semi-structured interviews & 11 focus group interviews	Thematic content analysis	9
Mauthner [61]	1995	18	Postpartum depression	Self report	England	Details not available	Semi-structured interviews	Voice centred relational method	5
Mauthner [62]**	1998	18	Postpartum depression	Self report	England	Local and national organisations, support groups, health clinics & ‘network sampling’	Semi-structured in-depth interviews	Voice centred relational method	6
Montgomery [63]**	2009	27	Perinatal depression	Self report	Canada	Peer support groups, community mental health agency, public health agency & family play centres	Unstructured interviews	Narrative analysis	7
Morrow [64]	2008	18	Postnatal depression	Self-report	Canada	General and family practitioners, community-based organisations,	Semi-structured interviews	Ethnographic narrative approach	8
Nahas [65]	1999	45	Postpartum depression	Self report	Australia	Arabic community centres	Unstructured interviews	Phenomenological study	9
O’Mahony [66]	2012	30	Postpartum depression	EPDS> = 10	Canada	Health care providers & organisations that provide mental health services	Semi-structured interviews	Critical ethnographic method	10
Raymond [67]	2009	9	Antenatal depression	Self report	England	GPs, nurseries, health visitors	Semi-structured interviews	Thematic analysis	9
Recto [68]	2020	20	Perinatal depression	Self report	USA	Parenting classes - school nurses and social workers helped recruit	Interviews	Deductive content analysis	7
Roseth [69]	2011	4	Postpartum depression	EPDS> = 13 or clinical diagnosis	Norway	Local healthcare clinic nurses or psych outpatient dept	Interviews	Descriptive-phenomenological method	6
Scrandis [70]	2005	10	Postpartum depression	Self report	Unknown	Community sites - churches, postpartum support groups, home visiting programs	Semi-structured interviews	Grounded theory	5
Shafiei [71]	2015	39	Postnatal depression	EPDS or self report	Australia	Antenatal clinics or postnatal wards	39 Semi-structured telephone interviews then 10 face-to-face in depth interviews	Thematic analysis	9
Tang [72]	2016	38	Postpartum depression	Self report	China	Convenience sample from community	Semi-structured interviews online	﻿Grounded theory approach and constant comparison method	6
Taylor [18]	2021	14	Perinatal depression	Self report	England	NHS health providers from primary care to acute and secondary mental health care	Semi-structured interviews	Social constructionist theory. Thematic analysis.	10
Templeton [73]	2003	20	Postnatal depression	EPDS> = 12	England	Local community known to health viitors	6 Semi-structured interviews + 14 from focus groups	Descriptive thematic analysis	6
Wittkowski [74]**	2012	10	Postnatal depression	EPDS> = 12	England	Health visitors and midwives.	Face to face interviews	Constant comparison and grounded theory approach	8

Key: EPDS = Edinburgh Postnatal Depression Scale; ** = Papers identified by hand searches; PND = postnatal depression; PPD = postpartum depression; NEET = Not in education, employment or training

The included data was coded by one researcher (KA) using NVivo 20, initially using line-by-line coding to search for concepts. Studies were coded in turn and subsequent studies were coded into pre-existing concepts, with new codes created when deemed necessary. An inductive data-led approach was then used to build up a framework of descriptive themes relating to the two core research questions with input from the wider research team. The descriptive themes were further compared and contrasted to create overarching analytical themes. The selected citations were then used to evidence the resulting thematic analysis and framework. A second researcher (CV) coded results data from 20% of included papers and this fed into the overarching discussion and selection of codes and themes.

### Patient involvement

A Patient Advisory Group (PAG) of women with lived experience of perinatal mental illness was vital to the design and conduct of this study. One member of the PAG provided feedback on the protocol, including shaping the initial research questions. A 1.5 hour online meeting was attended by 6 members of the PAG to discuss findings of the initial coding. The women drew on their own experiences of loneliness in the perinatal period and provided feedback on the initial findings and identified further potential emerging themes. Their feedback was instrumental in shaping the themes and identifying gaps in the data, providing validity to the overall thematic synthesis. A person with lived experience is also a co-author of this research paper – they were a member of the PAG who then read and commented on each draft and the paper was revised in response to this. In particular, she emphasised the importance of distinguishing between antenatal and postnatal depression; the difficulty women may have in identifying what can help them during their illness; and the importance of the baby in women’s overall experience. Overall, this shared experience helped gain a richer understanding of the material [37].

### Reflexivity

Quality standards in qualitative research require that authors consider how their own views and opinions could influence decisions made in the design and conduct of a study and influence emerging findings and vice versa [75]. Regular meetings were held with the wider multidisciplinary review team to check adherence to criteria, discuss methodology and seek their perspectives of emerging data and themes. The multidisciplinary team included academics and clinicians with a variety of perspectives and knowledge about loneliness, motherhood and perinatal depression based on their clinical, research and life experiences. The wider team consists of academics with experience and interest in the mental health impacts of loneliness, with some members conducting research on perinatal mental disorders. Some members of the team are also clinicians with experience of working with women with perinatal depression. They also have different personal experiences of motherhood.

KA is a white British, female MSc student in Mental Health Sciences Research and is a psychiatry trainee with a special interest in perinatal mental health and has experience of becoming a new mother during the pandemic. SJ is a psychiatrist with clinical and research experience relevant to the study topic, EP is an anthropologist with research expertise in loneliness, BLT is clinical psychologists and RN is an academic with a psychology background; both are researchers with research expertise in loneliness and perinatal mental health, CAW is a psychiatry trainee with clinical and research interests in perinatal mental health, CV is an MSc student in Clinical Mental Health Sciences with a psychology background, SS is a person with lived experience of perinatal mental illness.

## Results

### Description of included studies

**Fig. 1:**
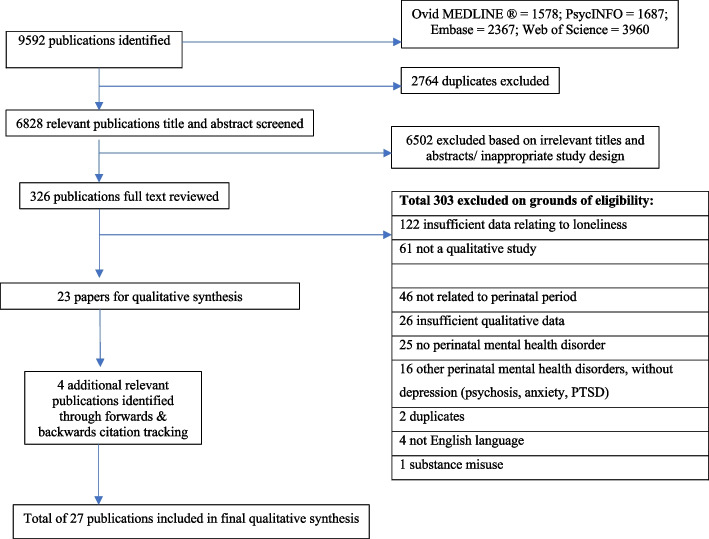
PRISMA Flow diagram for included studies (see additional pdf file)

Figure 1 presents a PRISMA flow chart demonstrating the selection process to retrieve the final included 27 papers from the initial 9592 records yielded by systematic searches. Quality scores based on the CASP quality assessment ranged from 4 to 10 out of 10, with a mean score of 7.4. The most common reason for lower quality scores was a lack of reflexivity in 63% of included papers. Brief characteristics and quality appraisal results of the included papers are summarized in Table 2 and Table 3 respectively. A further table (Additional file 2: Table 2) with comprehensive details of the included studies, including study aims, can be found in Additional file 2 (see Supplementary Materials).

**Table 3:** Evaluation of the quality of included studies according to the Critical Appraisal Skill Programme (CASP) Qualitative Checklist [48]

Study name & date	CASP Quality Assessment Score Categories										Total score
	Validity						Results			Value of research	
	Clear aims	Appropriate qual methodology	Appropriate research design	Appropriate recruitment	Appropriate data collection	Considered reflexivity	Ethics addressed	Rigor data analysis	Clear findings		
Beck 1992 [27]		✓	✓		✓		✓		✓	✓	**6**
Blanchard 2009 [51]	✓	✓	✓	✓	✓	✓		✓	✓	✓	**9**
Boath 2013 [52]	✓	✓	✓	✓	✓		✓	✓	✓	✓	**9**
Edhborg 2005 [53]	✓	✓	✓	✓	✓		✓	✓	✓	✓	**9**
Evans 2012 [54]	✓	✓	✓		✓			✓	✓	✓	**7**
Gardner 2014 [55]	✓	✓	✓		✓		✓	✓	✓	✓	**8**
Hanley 2006 [56]	✓	✓			✓		✓		✓	✓	**6**
Highet 2014 [28]	✓	✓	✓		✓		✓	✓	✓	✓	**8**
Jackson 2020 [57]	✓	✓	✓	✓	✓	✓	✓	✓	✓	✓	**10**
Keefe 2016 [58]		✓	✓						✓	✓	**4**
Keefe 2019 [59]	✓	✓			✓				✓	✓	**5**
Letourneau 2007 [60]	✓	✓	✓	✓	✓		✓	✓	✓	✓	**9**
Mauthner 1995 [61]	✓	✓				✓			✓	✓	**5**
Mauthner 1998 [62]	✓	✓			✓			✓	✓	✓	**6**
Montgomery 2009 [63]	✓	✓	✓				✓	✓	✓	✓	**7**
Morrow 2008 [64]	✓	✓	✓	✓	✓	✓			✓	✓	**8**
Nahas 1999 [65]	✓	✓	✓		✓	✓	✓	✓	✓	✓	**9**
O’Mahony 2012 [66]	✓	✓	✓	✓	✓	✓	✓	✓	✓	✓	**10**
Raymond 2009 [67]	✓	✓	✓	✓	✓		✓	✓	✓	✓	**9**
Recto 2020 [68]	✓	✓	✓		✓			✓	✓	✓	**7**
Roseth 2011 [69]		✓	✓			✓	✓	✓	✓		**6**
Scrandis 2005 [70]		✓		✓				✓	✓	✓	**5**
Shafiei 2015 [71]	✓	✓	✓	✓		✓	✓	✓	✓	✓	**9**
Tang 2016 [72]	✓	✓	✓					✓	✓	✓	**6**
Taylor 2021 [18]	✓	✓	✓	✓	✓	✓	✓	✓	✓	✓	**10**
Templeton 2003 [73]	✓	✓	✓		✓		✓			✓	**6**
Wittkowski 2012 [74]	✓	✓	✓			✓	✓	✓	✓	✓	**8**

The included papers were published between 1992 and 2021. All studies were conducted in high or upper-middle income countries, as defined by the World Bank [76]: UK (*n* = 10), USA (*n* = 6), Canada (*n* = 5), Australia (*n* = 3), Sweden (n = 1), Norway (n = 1) and China (n = 1). Eleven (41%) papers exclusively explored the experiences of women from different marginalised groups (including immigrant (*n* = 4), ethnic minority (n = 6) and teenage mothers (n = 1)). The majority of the studies involved women who had experienced depression after birth (postnatal, *n* = 21; antenatal, n = 2; entire perinatal period, n = 4). Most studies included women with self-reported depression, although 9 papers did use the Edinburgh Postnatal Depression Scale (EPDS) to confirm their diagnosis using scores of equal to or greater than 10, 12 or 13. Only one paper had an explicit research aim to explore the experience of loneliness in perinatal depression [18]. The remainder of papers had a broader aim to explore the availability of social support for women with perinatal depression or to more generally explore the experience of perinatal depression. Study participants were typically recruited from primary healthcare or community settings, such as antenatal groups. The majority of studies (*n* = 26, 96%) involved data collection through individual face-to-face or telephone interviews – using either semi-structured or unstructured formats - and/or focus groups. One study exploring content on an online postpartum depression support website used individual online messages as the unit of analysis. A range of different methodological approaches for analysis were used, including grounded theory, phenomenological and ethnographic studies.

Together the studies represent the views of more than 537 women, with a mean age of 27.7 years (across the 12 studies in which age was reported) and ages ranging from 15 to 49 years. Study sample sizes ranged from 4 to 45 participants. They represented women from a limited range of ethnic and cultural backgrounds and socio-demographic groups. Women with different marital and co-habitation statuses and both primiparous and multiparous women were included.

### Thematic synthesis

**Fig. 2:**
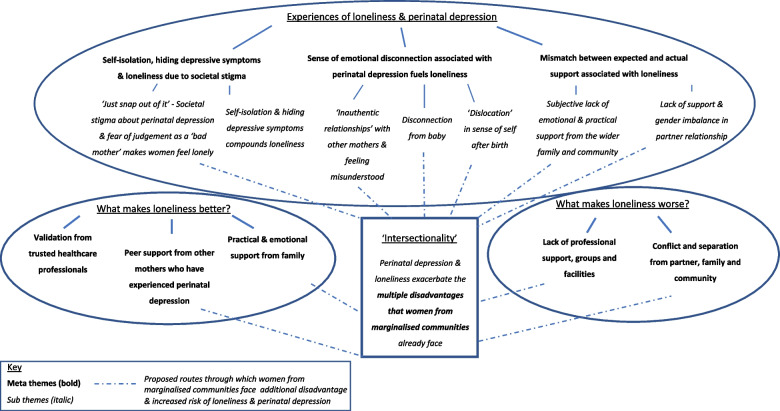
Map of meta-themes and sub-themes by research question (see additional pdf file)

Through the thematic synthesis, we identified eight overarching descriptive meta-themes and three sub-themes focused under three headings: ‘Experiences of perinatal depression and loneliness’; ‘What makes loneliness better for women with perinatal depression?’; and ‘What makes loneliness worse for women with perinatal depression?’. Fig. 2 is a pictorial representation of these themes.

Throughout the studies women described feelings of loneliness or feeling alone, providing data with a clear and obvious link to the research question. Many women also discussed feelings of disconnection, a lack of sense of belonging or a perceived lack of support - these experiences were only included in the analysis if there was a clear connection with the concept of loneliness.

Table 4 presents additional primary data to support each section of the thematic synthesis and Additional file 3 (see Supplementary Materials) presents a table (Additional file 3: Table 3) with further comprehensive supporting primary data. Primary quotes from women in the studies are depicted in quotations and *italics* and secondary interpretation from the included studies is presented in quotations.

**Table 4:** Meta-themes, sub-themes and primary supporting data

Research question	Meta-theme	Sub-theme	Primary data
Experiences of loneliness and perinatal depression	1. Self-isolation, hiding depressive symptoms and loneliness due societal stigma	*1.1 ‘Just snap out of it’ – Societal stigma about perinatal depression and fear of judgement as a ‘bad mother’ makes women feel lonely*	‘It was clear from women’s narratives that many experienced a sense of failure or inadequacy that could prevent them from connecting to others. The mothers described feeling under pressure—from themselves, partners, family members, other mothers, and wider societal narratives—to take on the role of a primary caregiver and to be, as Lottie put it, “a perfect mum”’ [18]
		*1.2 Self-isolation & hiding depressive symptoms compounds loneliness*	‘The participants described a withdrawal from others by hiding their true thoughts and feelings by isolating themselves. One mother described vividly how her strong feelings of shame made her actively conceal her real feeling and thoughts. Another mother described how she exhausted herself by putting on a happy mask and doing her best to keep up appearances’ [69] ‘There was also a tendency in depressed women to isolate themselves. Women described a strong desire to not have to leave the house but rather be alone and for others to go away: “*I didn’t want to see anyone – even though I needed support I just wanted everyone to leave me alone”* [28]
	2. Sense of emotional disconnection associated with perinatal depression fuels loneliness	2.1 ‘*Inauthentic relationships’ with other mothers and feeling misunderstood*	“*This girl I knew. .. I said “Oh, do you feel like that, do you?” and she’d say “Oh, no, no, no” and I said “Oh-oh, pull yourself together dummy. .. you’re alright.” and then I’d get home and think “She doesn’t feel like that, perhaps it isn’t normal”. ..*. *That was the one thing that really got to me through it all, that I couldn’t find anyone who felt like I did, and I felt like I was going through it on my own. ... I couldn’t find anyone who said “Oh yes, I felt like that, don’t worry, you’ll get better”. ... I felt really isolated and lonely through it*.’ (Pam)’ [61]
		*2.2 Disconnection from baby*	“Several women also described difficulties bonding with their babies, for example feeling “nothing”, “numb”, “terrified of them” or like their baby “wasn’t a part of me”. For some mothers, a baby’s need to be close to them could feel uncomfortable, even threatening, yet a lack of closeness also resulted in high levels of distress. As Emma explained: “[*My baby] used to have to sleep on my chest and, because I didn’t want him near me, it was really hard having him on my chest. So he used to just lay there and scream in pain and I used to just sit in another part of the room and just cry*.”’ [18]
		2.3 *‘Dislocation’ in sense of self after birth*	‘….feelings of dislocated identity clearly included a strong sense of loneliness and desolation, as the mothers described themselves becoming confined to their homes with their babies, isolated from the wider world, and disconnected from their past lives and social networks. A dislocated self was particularly evident in the narratives of first-time mothers who had stopped working or taken maternity leave to have a baby.’ [18]
	3. Mismatch between expected and actual support associated with loneliness	3.1 *Subjective lack of emotional and practical support from the wider family and community*	‘Anna left a safe, secure social network back home to find a very precarious situation in her new country: *The biggest problem that I faced was arriving late in my pregnancy and didn’t have a specialist. So when it’s time for delivery they said go to the ER. .. I was so stressed the words were gone and was unable to speak. Everything is new. .. you are alone and not with your family and don’t know their system, so this is scary.. .*’ [66]
		3.2 *Lack of support and gender imbalance in partner relationship*	‘Women spoke about feeling isolated and alone and overwhelmed with anxiety by being the sole caregiver for their infants while their partner was at work.’ [60]
What made loneliness better for women with perinatal depression?	4. Validation from trusted healthcare professionals		*I recognise she can only come to the house once a week and then only for 2 hours but they are the two most important hours in my week. My volunteer is more like a friend than a person in a ‘working relationship’* [56]
	5. Peer support from other mothers with perinatal depression		‘*Groups are a safe place to say ‘This isn’t the greatest time of my life’, and getting some support that you are a good mom, and that your baby does feel loved, even though you’re not, like, jumping up and down for joy, and that it will get better*.’ [60] ‘All participants within this study were recruited from mother and baby groups. For those mothers who were feeling particularly isolated, these groups provided not only a source of support and knowledge but also gave women a sense of community, which is embedded within collectivist West African culture: ……… [*when you start going to the group] you know that you are not alone. So many mothers are going through what you are going through. And some are even MORE than yourself…….. [I think] there should be a gathering for mothers……. So you can chat with another mother.…. it does help*. – Participant 1’ [55]
	6. Practical and emotional support from family		‘Health services often did not accommodate women’s children during treatment sessions, and therefore some women relied on informal childcare to enable them to access this formal support*. I’ve been having counselling every week and I’ve come on in leaps and bounds… my Mum and Dad kept these two (children) and it’s so helpful*. (Tina, postnatal depression and post-traumatic stress disorder, rural village)’ [57]
What made loneliness worse for women with perinatal depression?	7. Lack of professional support, groups and facilities		“*There’s nothing really in this area for mothers. I found it very difficult to meet up locally. ... I would have liked to have known. .. other mothers around, and if there was sort of a central meeting place in this area’* [61]
	8. Conflict and separation from partner, family & community		‘Most participants expressed that being a newly immigrated mother without the familiar, preexisting support networks could predispose women to PPD. Women felt that they were vulnerable because of the lack of family support after childbirth. For Anna, being connected was the norm back home: *“The families are so big and so supportive. We’re always in touch. .. always gathering around someone.*” Kate maintained that PPD is more commonly found here because of the lack of familial support and the stress of being alone. She felt that isolation and solitude are the biggest problems in developed countries.’ [66]

### Experiences of loneliness and perinatal depression

#### Meta-theme 1 – self-isolation, hiding depressive symptoms and loneliness due to societal stigma

Sub-theme 1.1 - ‘Just snap out of it’ - societal stigma about perinatal depression & fear of judgement as a ‘bad mother’ makes women feel lonely

A key theme that emerged in the majority of studies was the deep and interwoven sense of shame that women felt about their depression and their fears of being judged as a ‘bad mother’ and ‘bad wife’ [18, 52, 53, 64, 65]. There was a sense that they were expected to be happy and relishing their role as a new mother. They strived to be the ‘perfect mum’ that society expected but the pain of their depression and resulting stigmatisation left them feeling like a failure and lonely (‘It was clear from women’s narratives that many experienced a sense of failure or inadequacy that could prevent them from connecting to others’ [18]).

Mothers experienced criticism, rejection or discrimination related to both their depression and new motherhood from many different sources: from partners, family, friends, healthcare professionals, colleagues and sometimes even themselves [27, 57, 60, 61, 63, 72]. A few women described direct, explicit experienced stigma (‘the mothers were told to ‘*just snap out of it. You have a husband who loves you and a beautiful healthy baby. What more do you want!”* [27]), whilst many more described less-explicit, self-perceived sources of criticism or even self-judgement.

‘*And my husband would always really try to tell me to be more cheerful, but it would always seem impossible and of course it added to more guilt on my part*’ [64].

Women seemed particularly affected by societal pressures around breastfeeding, which could be intensified by healthcare professionals [53, 72], a theme that was emphasised by the Patient Advisory Group.

Stigma seemed to be particularly acutely felt amongst socially disadvantaged groups - such as immigrants, ethnic minorities or adolescent mothers – who felt a double burden of discrimination based on both their mental health and their minority status that complicated their pre-existing social isolation further [18, 74] (*‘LD: I hid it for months. SA: You feel that they might try and take your baby off you because you’re young*.’ [52]; ‘*There is a huge stigma of being mentally ill in the public, but for us Asians there is a double disadvantage*’ [74]).

Sub-theme 1.2 – self-isolation and hiding depressive symptoms compounds loneliness

Women seemed to find the constant fear of negative judgement made it increasingly painful to be around other people – particularly family, friends and other mothers – and women reported the need to withdraw and self-isolate from others to protect themselves from these uncomfortable feelings. This self-enforced isolation led to deep feelings of loneliness and disconnection (‘*It’s just like you are an island on your own. I have got nobody to help me.*’ [28, 55, 69]).

As a result of this perceived stigma, women would also try to minimise or hide symptoms of depression from others, including healthcare professionals [53, 60]. They would not seek help despite recognising their need for it – seemingly due to a perceived taboo about asking for help and a sense that they should be coping - and this could lead to delayed diagnoses and increased feelings of loneliness (‘... *it took me about 9 months to finally seek help from the doctor’* [63]).

‘*what comes out of your mouth is, ‘ No, I don’t need any help ’ even though inside, you’re screaming, and saying, ‘Yes, for God’s sake, help me.”* [60].

Hiding symptoms could also be due to an increased fear of social services involvement or even potential child removal due to their depression (‘*I just lied through my teeth because I thought, “What are they going to do if they find out I can’t be a good mom?*”’ [60]).

#### Meta-theme 2 – sense of emotional disconnection associated with perinatal depression fuels loneliness

The experience of depression at the same time as the arrival of a new baby made it difficult to sometimes untangle one single underlying cause of women’s loneliness. Even women with good support networks described an inability to emotionally connect with others – their peers, baby and themselves – which they couldn’t understand or explain (‘*Although I had a lot of support from a lot of people, which should have made me ecstatic, I couldn’t actually connect with it*’) [18, 27, 67].

Roseth et al. described: ‘After the birth, the mother experienced sudden and repetitive lapses into intense feelings of unreality and disconnection both in regard to self, the baby, and the social and material world’ [69].

Sub-theme 2.1 - ‘inauthentic relationships’ with other mothers and feeling misunderstood

Many women spoke about their difficulties in connecting with other mothers. They spoke of a desperate need to reach out and hear from women who were going through similar experiences, particularly those with perinatal depression. Yet they felt like ‘frauds’ during these interactions *(‘It’s almost like being a*. *.. pretender. .. . I’m putting across what I think people want to see rather than what I am myself’* [61]). They were unable to share their true experiences due to the shame of feeling ‘different’ due to their depression, yet at the same time were internally comparing themselves with everything the other mothers were saying [61]. These interactions felt inauthentic and often served to undermine women’s confidence further and made them feel lonelier in their predicament [18].

Women also reported a sense that no one understood what they were experiencing or the reality of their experiences – this could be other mothers, their friends or their partners and families. There was a sense that they were the ‘only one’ going through this, which heightened their sense of loneliness (‘*That was the one thing that really got to me through it all, that I couldn’t find anyone who felt like I did, and I felt like I was going through it on my own’* [61]).

Often antenatal groups and mum and baby groups - where these constant comparisons were perceived as unavoidable - were the only social activities that were available to pregnant and new mothers. However, if women didn’t attend the groups to avoid these interactions, they would end up feeling even more socially isolated and alone, creating a vicious cycle [61]. This theme was particularly emphasised by the Patient Advisory group.

Sub theme 2.2 – disconnection from baby

Difficulty bonding and negative emotions towards their baby are both common symptoms of perinatal depression [77]. Feelings of disconnection from the baby were linked to feelings of shame and loneliness, particularly due to the high expectations of an immediate mother-infant bond (‘*I just expected to fall in love with him totally, straight away, which I didn’t … I hate myself for that’* [62]. Many women spoke about a sense of emotional aloneness that was almost exacerbated by having to be in the constant presence of their baby (‘[*My baby] used to have to sleep on my chest and, because I didn’t want him near me, it was really hard having him on my chest. So he used to just lay there and scream in pain and I used to just sit in another part of the room and just cry’* [18]). For some this seemed to be particularly pronounced at night times (‘“*It’s just. .. later on in the daytime when the doors close. .. it’s hard, like. .. at night time. .. where you all alone.*”’ [59]).

Sub theme 2.3 - ‘dislocation’ in sense of self

In many papers, women spoke about a loss of identity or a ‘dislocation in their sense of self’ [18]  resulting from both their transition to motherhood and their experience of depression. Many spoke about the loss of their work and old friendship networks (‘At work, some mothers felt they could not share their new experiences of parenthood with colleagues who were not parents themselves. Several mothers felt alienated from colleagues who disapproved of working mothers’ [61]) or their loss of skills and confidence, their lack of mastery in their new role as a mother or even their lack of body confidence (“*Before, I was so confident and outgoing and it was like, ‘Hey, where’s the party?’ And now, it’s just, you just feel lost, in a way’* [18]). Whilst this role transition is ubiquitous for all new mothers, the loss of sense of self seemed to be a recurrent narrative for women with perinatal depression and seemed to be linked to a sense of aloneness that they would never recover their old selves (“*My big fear was that I wasn’t going to get better and even if I got better that I wasn’t going to be the same person I was before the experience*” [27]; ‘She struggled to feel connected, integrated and capable at home with her baby, no longer having a routine or role in which she felt competent’ [18]).

Some women interpreted this loss of self in relation to the new focus on the baby (feeling like a ‘walking womb’ or a ‘baby carrier’ [67]) and feeling that their needs were no longer noticed [52]. Others related this loss of self to the inability to enjoy life in the way that they used to, partly because the relentlessness of motherhood left them no time for themselves [18, 27, 28] (“*I felt as if I was acting. I went through the motions of my life without any of the joy*” [27]).

#### Meta-theme 3- mismatch between expected and actual support associated with loneliness

Sub theme 3.1 – subjective lack of emotional and practical support from the wider family and community

In nearly all studies, women spoke about a mismatch between the support they expected and needed from their partner, family and community and the actual support they were offered, leading to feelings of loneliness [55, 57, 59, 64, 72] - ‘*I expected my mother-in-law to come and help with things around the house, but she refused. I was very upset ….*’ [72]. Women seemed to feel particularly lonely when caring for their child alone, such as when their partner went out to work, and felt their partner did not appreciate how difficult this was [56, 57, 60, 63].

A subjective feeling of loneliness could exist even if objectively they could identify there was a lot of support available – in some cases mothers identified that the ‘right kind’ of support was not being offered (e.g. practical support instead of emotional support) and in other cases due to their depression they felt less able to ‘*connect*’ with the support being offered (as described in quote above).

Women distinguished between different types of support – both emotional and instrumental. A lack of emotional support seemed to be more often linked to the feeling of loneliness (*“*Company’ for her was about talking, listening and being emotionally available and not just being physically present in the room*’* [55]). Women felt they needed more support because of their struggles with postnatal depression [72], although many described not asking for support due to the fear of being ‘a bother’ [56]. Similar feelings of loneliness were described by women who were physically, culturally or geographically isolated and lacking the emotional support that they felt they needed – whether due to rural location, immigration or perceived lack of safety in their neighbourhood [55, 57, 59, 71].

Sub theme 3.2 – lack of support and gender imbalance in partner relationship

Lack of support from a partner or co-parent was an important theme in the majority of papers. Women seemed to feel their depression particularly impacted on their ability to connect and feel close to their partners and vice versa. There was also a loss of important elements of their relationships with their partners, such as time for mutual interests or intimacy (‘” *I just wanted him to leave me alone and not touch me*”.’ [27]) [53]. New parenthood could also amplify the gender imbalance in these partner relationships, particularly around caring responsibilities, which could leave mothers feeling lonely (‘Women spoke about feeling isolated and alone and overwhelmed with anxiety by being the sole caregiver for their infants while their partner was at work’ [60]). They would strive to keep the relationship going due to their feelings of vulnerability with a new baby and fear of being left alone, yet this lack of support often led to increased feelings of loneliness [18, 68].

‘*I started feeling more depressed during my pregnancy because my significant other was like, “Oh, just have an abortion …” and I just didn’t feel his support’* [68].

### What made loneliness better for women with perinatal depression?

#### Meta-theme 4 - validation from trusted healthcare professionals

Women described the importance of their symptoms being recognised by health and social care professionals and being given a diagnosis (or ‘permission to be ill” [56]) that could validate their experiences. Many women felt that healthcare professionals were the only ones they could trust (*‘I trust her (health care provider) and I’m completely comfortable with her’* [68]) and there was a sense that professionals had an important role in helping these women feel less emotionally lonely in their perinatal depression, sometimes even feeling ‘kind of like family’ [18, 52, 56, 68].

Women emphasised the importance of one-to-one support, continuity of care or access to regular support in their own home, particularly in the early days following birth [52]. Community support by peer volunteers (such as Home Start in the UK) and home visits by midwives were examples of other health and social care interventions that women credited with making them feel more connected and providing emotional support that could make them feel less lonely [56]. This theme was strongly emphasised by the Patient Advisory Group.

#### Meta-theme 5 - peer support from other mothers who have experienced perinatal depression

To overcome the experience of loneliness relating specifically to the sense of having ‘inauthentic relationships’, women described the importance of being able to talk to other mothers that they felt empathised and understood their situation – particularly other mothers experiencing postpartum depression. These connections were valued both in person or online [18, 54, 55, 60, 67]. They felt that peers who had experienced perinatal depression too were less likely to judge and found the opportunity to share their experiences with these women liberating and helped them to feel less lonely, particularly in the setting of support groups and if they shared a similar cultural background [55, 60, 70] (‘*It was very comforting to listen to them tell me that they understood … and that it was going to get better … and that I wasn’t a bad mother*’ [60]).

#### Meta- theme 6 – practical and emotional support from family

Practical support from their partners and wider family, particularly with childcare, seemed to help with women’s loneliness [18, 57, 60, 68]. Having some space away from their babies and ‘alone time’ paradoxically helped them feel less lonely by connecting with their old and new selves or enabling them to access treatment for their perinatal depression (‘*just taking the baby out so I can have some alone time … ’* [63]).

Women also felt less lonely if they felt their partner and family understood and appreciated their diagnosis of perinatal depression and offered emotional support [60]. Despite the transition to their new mother role, many women described still wanting to be ‘mothered’ themselves (“*I just wanted to be looked after*” [18]) and particularly valued support from their mothers or female family members [60].

### What made loneliness worse for women with perinatal depression?

#### Meta-theme 7 - lack of professional support, groups and facilities

Lack of professional validation would sometimes lead women to minimise their own depressive symptoms and often made them feel that they needed to cope alone [56] (‘*I felt that it wasn’t my place to talk about my feelings. It was not welcome there … so I just preferred not to say anything*.’ [66]). Women felt particularly upset and lonely if professionals asked questions in a disinterested, ‘tick box’ way, if professionals minimised their symptoms, for example, saying that they were part of ‘normal motherhood’, or if they had to see many different professionals or people they did not feel comfortable with [56, 60, 63, 68]. An abrupt end in professional support could leave women feeling lost and emotionally alone (‘“*Then my midwife stopped and she was kind of like, like somebody that I really relied on … it made me feel, I don’t know, like I’d had a loss*.” [18]).

Even if women did try to access support, there was often a lack of appropriate spaces or services for mothers with perinatal depression – whether this was physical spaces suitable for mothers and babies to meet, or a lack of appropriate support groups specifically for perinatal depression [57, 60, 61, 66, 70, 73]. Some women described experiencing language and cultural barriers when trying to access healthcare and a lack of familiarity with the new system they operated in, others described experiencing overt racism [66, 71, 74], all of which exacerbated feelings of loneliness and helplessness [66].

‘*My husband left me in pregnancy, and I have no-body, my family are in India. I can’t speak English properly, and I can’t read English to fill out forms. I need help, don’t know where to go, or who to turn to’* [74].

#### Meta-theme 8 –- conflict and separation from partner, family and community

The period of pregnancy and post birth was identified as putting a new and unique strain on existing family and partner relationships and this was exacerbated by a diagnosis of perinatal depression. If there was additional stress on a relationship - such as domestic abuse or substance use or separation due to work or immigration - then the relationships became even harder to navigate for women and increased their feelings of loneliness and isolation [55, 59, 65, 73].

Feelings of loneliness seemed to be particularly strong in women separated from their family and cultural support systems– for example, women who were immigrants. (“*You feel like you’re alone in the world, there is not many people to care for me, and I don’t have family here … I felt lonely”)* [55, 71]. Women seemed to particularly mourn the separation from cultural traditions that were overtly supportive and offered companionship in early motherhood and many felt that they wouldn’t have been lonely or depressed if they were living within their own country or community of origin [55, 66].

‘*You are attached to the family house you are not on your own, they make meals for you … the pressure is very low in Africa, it is not like that here.*’ [55].

## Discussion

### Main findings

This meta-synthesis of qualitative studies that contained women’s accounts of loneliness in relation to their perinatal depression identified several important themes. Women’s tendency to self-isolate and hide their symptoms due to both self-perceived and experienced societal stigma was linked to loneliness. Loneliness was experienced as a sense of emotional disconnection from self, baby and other mothers following the transition to motherhood. There was often a mismatch between expected and actual family support – particularly affecting partner relationships – that exacerbated loneliness.

Women with perinatal depression felt that validation and understanding from trusted healthcare professionals, peer support from other mothers with perinatal depression, and both practical and emotional support from family were all important factors that could make the experience of perinatal depression and loneliness better. There was a sense that women felt lonelier when professionals minimised their symptoms or there was an absence of appropriate peer support groups. Many women with perinatal depression described how conflict and separation with partners, family and community – particularly separation from their own cultural communities - could lead them to feeling more lonely.

### Findings in context of other studies

Existing qualitative and quantitative studies have already demonstrated an association between depression and loneliness and between parenthood and loneliness [13, 22]. However, this study suggests there is also a unique interaction between perinatal depression and loneliness due to the particular circumstances and stressors associated with the transition to motherhood and the increased risk of depression at this time. This study also builds on the existing available evidence about perinatal depression. Previous qualitative studies looking at broader social experiences of women with perinatal depression (rather than loneliness specifically) found women described frequent experiences of loneliness [27–29]. One recent qualitative study specifically exploring loneliness in perinatal depression (that was included in this meta-synthesis) found women repeatedly described feeling lost, alone and isolated and linked these feelings to a lack of supportive relationships, fears of judgement and dislocation from their previous lives and identities [18]. These themes were strongly supported across the studies included in this meta-analysis.

The findings in this meta-synthesis support Weiss’ broader conceptualisation of loneliness as consisting of both ‘emotional’ and ‘social’ loneliness [10]. Women described feelings of being lonely, isolated, alone, disconnected in relation to a lack of intimate attachment and inability to confide in specific other people (partner, peers, family and even self) and also a lack of belonging to a wider social group or network that resulted from not feeling like a ‘good enough mother’ (such as the lack of authentic connections with other mother peers and lack of belonging in support groups). ‘Existential’ loneliness seemed to be described less commonly as a theme for these women with perinatal depression, although perhaps could be linked to the ‘disconnection’ some women described between their old and new sense of self and identity.

Many women described how the lack of expected support from family and the wider community had triggered or amplified their depression and impacted their feelings of loneliness. Many other women talked about how common symptoms or experiences associated with perinatal depression – inability to bond with their baby, fear and guilt at being a ‘bad mother’, inability to enjoy themselves – lead them to feel even more lonely. This interconnected relationship between perinatal depression and loneliness supports previous research findings that loneliness can be both a cause and a consequence of depression and the two experiences can be bidirectional and mutually reinforcing [40].

Moreover, previous research has found that loneliness is an important mediator in the relationship between mental health-related stigma and depression [78]. Self-stigma and experienced stigma were both dominant themes in the studies included in this synthesis and many women described an enforced self-withdrawal and isolation to avoid actual or perceived judgement and discrimination. This is reflected by this study’s title – one woman reported being told to ‘just snap out of it’ and related this negative interaction related to her depression to an increased sense of loneliness and isolation.

For some women a lack of family support or health care service provision resulted in an objective sense of aloneness that was not accompanied by an emotional aloneness – but many women reported experiencing both. There were also many women who did have access to practical and emotional support but did not feel able to connect with it and felt lonely in spite of it.

Many previous studies have focussed on the social experiences of more marginalised groups with perinatal depression (such as teenage, ethnic minority and immigrant mothers) and find that loneliness is complicated by the multiple disadvantages that these women face [18, 29–31, 35]. In keeping with these previous studies, this review found an important intersectionality between stigma and minority group status, whereby women from marginalised communities described how perinatal depression exacerbated the inequalities that they experienced and lead them to retreat further from people and places where they might otherwise have found connection. The social context of motherhood in these disadvantaged groups (for example, the lack of extended family networks for immigrant mothers) combined with their pre-existing lack of integration into society reinforced feelings of loneliness for these mothers.

The testimony of women about their experience of a mismatch in the expected and actual support they received resonates with Mauthner’s relational theory of postnatal depression – the disconnection between expectations of a ‘universally happy time’ and the reality of women feeling inadequate and unsupported - as well as Peplau’s definition of loneliness [9, 79]. Mauthner also described a vicious cycle of ‘self silencing’ and withdrawal that was reflected in the accounts of women in this meta-synthesis who felt a ‘double bind’ of loneliness – needing more support due to their depression but getting less support because of it [80].

Social identity theory states that social groups and relations are an integral part of one’s identity. Evidence suggests that individuals who identify highly with a group tend to report less depression [81]. Some theories propose that loneliness can be normative during important transitions due to a lack of others experiencing the same transition, a loss of roles or change in social networks [82]. This study complements feminist literature that proposes the transition to motherhood can result in a more complex and pathological sense of ‘loss’ of identity and sense of belonging and inequality for women, including feelings of bereavement; loss of career, status and confidence; and a ‘dislocation’ from their former selves [28, 79, 83–88] – and also demonstrates how perinatal depression can further pathologise this more normative experience of loneliness.

Research has shown that people’s sense of belongingness to, and identification with, social groups has important health and wellbeing benefits and community-level interventions that enhance community identification and peer support can improve feelings of loneliness and depression (for example, Groups4Health [89]) [81, 90–92]. Existing studies exploring peer support in the perinatal period in the UK and North America have demonstrated both acceptability to mothers [93] and a reduction of scores for mothers on the Edinburgh Postnatal Depression Scale, whether they engage with online [94], telephone-based [95], group-based [96] or one-to-one [97] peer support [98]. However, data relating to peer support groups was one area of contradiction in this study. Most women seemed to feel that attending support groups and connecting with like-minded mothers could help them feel less alone. But some women found groups exacerbated their feelings of inauthenticity and loneliness, depending on the characteristics of other attendees – particularly if they felt misunderstood by these peers. These conflicting experiences need further exploration to inform the development of more accessible peer support interventions specifically for women with perinatal depression.

This study identified an important role for healthcare professionals in influencing women’s experience of loneliness and perinatal depression. The difficulty and fear of sharing their depression symptoms with healthcare professionals seemed to increase loneliness and delay appropriate treatment for women’s perinatal depression. However, many women in this meta-synthesis also described how sharing their experiences with a trusted healthcare professional helped them feel less lonely. Indeed, loneliness has been found to increase certain types of health and social care utilisation in older populations [99]. In keeping with research from other mental health settings, women also reported a sense of loss and aloneness when professional support ended [100], indicating the importance of trusted and timely health care interactions with clarity around nature and duration of interventions.

### Strengths and limitations

This qualitative meta-synthesis builds on existing research by combining available primary data to answer a novel research question about which factors might alleviate or worsen loneliness in perinatal depression – providing answers with the potential to positively impact clinical practice and the lives of women with perinatal depression.

The study used a well-recognised and robust methodology and systematic search strategy that was pre-registered and supported by a team of experienced researchers [37]. Issues of reflexivity and threats to validity were acknowledged and explored through regular meetings of the multidisciplinary review team. The involvement of women with lived experience throughout the project was vital for improving the robustness, validity and clinical utility of the results.

Social isolation and loneliness are seen increasingly as priorities when supporting people with mental health difficulties [21, 40]. Given pregnancy and the postnatal period are times of increased health and social care contact, the peripartum could serve as an opportune moment to intervene to prevent or alleviate depression, particularly given the recent increase in investment in National Health Service perinatal mental health services in the UK [101, 102]. The findings are highly relevant and particularly timely given the significant and disproportionate impact the recent Covid-19 pandemic-related health and social restrictions (such as absence of support during labour and early childrearing and lack of face-to-face access to midwives, health visitors and support groups) seem to have had on perinatal women’s mental health both in the UK and globally [103, 104].

There are some limitations in this review that relate to limitations in the existing evidence examining loneliness and postnatal depression qualitatively. In terms of the quality of included studies, 63% neglected to address issues of reflexivity in the original paper so the subjectivity of findings must be kept in mind when interpreting the results.

A key limitation of this study is that only one included study explored loneliness experiences in women with perinatal depression as a primary aim, the remaining studies had broader aims about experiences of perinatal depression or social support more generally. This meant that the included studies did not necessarily probe the experience of loneliness – for example, as far as we are aware none of the participants across the 27 studies were specifically asked what makes feelings of loneliness better or worse for them. This reduces the richness and variety in the available data relevant to this study’s research questions. However, it does highlight the importance of loneliness for these women as loneliness experiences were raised spontaneously in all of these studies.

There was some heterogeneity in the method of diagnosis of perinatal depression and many women retrospectively self-reported their depression and experiences, thus introducing recall bias and reducing the overall diagnostic validity of perinatal depression across the included studies. The majority of papers were reporting experiences of postnatal depression, so the overall results tell us more about experiences of loneliness after birth than during pregnancy. It would be interesting to explore in future research how the different stages of the perinatal period and the transition to motherhood impacted on both women’s experience of loneliness and perinatal depression.

In terms of the overall study methodology, any meta-synthesis involves re-analysis of previously analysed data and could lead to superficial interpretations that undermine the integrity of the original primary research. Meta-synthesis also decontextualises the primary data, removing the ability to interpret the original accounts in the context of their location, time or the research studies specific analytic perspectives for example [38, 42]. Given there were 27 studies in this synthesis with heterogeneous analytic approaches, this is a particular concern. The efforts made at convening a Patient Advisory Group meeting and regular researcher meetings were a reasonable attempt to address reflexivity and provide more confidence about the interpretations. There was also heterogeneity in the study populations, type of perinatal depression, comorbidities so it is difficult to generalise the findings to individual patients.

This subjectivity in the selection of primary data may have been greater given that the initial search used not only the term loneliness, but also a range of closely related but distinct concepts (such as social isolation, support, connectedness). It is possible that some included quotes may be referring to these closely related concepts rather than loneliness per se, thus may not be appropriate to compare or combine thematically [9, 10].

This study excluded studies related to specific interventions, which limits the ability to draw conclusions about how different types of formal support might impact on women’s loneliness experiences and their perinatal depression. For example, there is a lack of data about how different types of peer support (online vs face-to-face; group vs individual) might improve or ameliorate loneliness.

The majority of studies contributing to this meta-synthesis were conducted in high income countries in the Global North, with relatively homogenous socio-cultural settings, making it difficult to speculate about the impact of different cultural contexts. The experiences of women in the one study based in a ‘non-Western’ country that described the impact of a unique Chinese perinatal cultural ritual (‘doing the month’ - involving one month of confinement after birth [72]) in fact mapped well to the overarching meta-synthesis theme of mismatch in expected and perceived support – suggesting that there may be similarities cross-culturally in loneliness experiences in perinatal depression. However, more research is needed into interpretations of both perinatal depression and loneliness experiences in a range of cultural and social contexts [105].

### Clinical and policy implications

This study provides important insights for clinicians, women and their families, peers and researchers. Lay dissemination of these findings could help women feel more understood and less alone in their experiences of perinatal depression and could educate others about how to support them to feel less lonely during their transition into motherhood.

Given the centrality of loneliness in the accounts of women’s experiences of perinatal depression, it seems appropriate that perinatal women are asked directly both about symptoms of depression and feelings of loneliness when they are accessing healthcare - particularly maternity services - to assess whether they are at risk of perinatal depression or indeed whether their loneliness is exacerbating any existing depression. Other key suggestions to clinicians could be (i) to explore and validate women’s experiences of perinatal depression, (ii) encourage women to share their diagnosis with trusted family and (iii) signpost to peer support from other mothers with lived experience of perinatal depression. Indeed, these findings emphasise the importance for all people interacting with perinatal women to reassure them that they are a ‘good enough’ mother where possible. These results also suggest a need for greater access to support groups specifically for women with perinatal depression that are well facilitated and held in accessible spaces, including online forums.

Local and national health and social care services need to think about the perinatal experiences of women in marginalised communities and consider how their unique experiences of new motherhood, social isolation, discrimination and perceived stigma put them at increased risk of experiencing loneliness and developing mental health complications. Providing clear access to healthcare and social support that is culturally appropriate and without language barriers is a very basic first step in meeting the complex needs of these women and helping them feel less alone. Interventions and campaigns that attempt to reduce perinatal depression-related stigma both in these communities and across the general population, whether run by public health bodies or third sector organisations, are urgently needed.

### Future research

To address some of the limitations raised in this study and develop a more comprehensive understanding, it is important to conduct more primary qualitative interview studies with a specific focus on women’s experiences of loneliness and perinatal depression, both in pregnancy and after birth. Further research using quantitative techniques to empirically test the relationship between perinatal depression and loneliness is also required.

More qualitative research is particularly needed in low- and middle-income countries to gain an understanding of how resource poor settings and different healthcare systems and cultural contexts impact upon women’s experience of loneliness and perinatal depression. Further exploration of loneliness and perinatal depression in other marginalised groups is required given the double burden of stigma they experience, for example, women who experience domestic abuse or use substances. Future research could also attempt to unpick the degree to which loneliness experiences are associated with all perinatal mental health disorders rather than just depression - as we found a relative lack of qualitative research for other psychiatric conditions - and the impact of physical health obstetric complications on loneliness – as this was an area highlighted by the Patient Advisory Group that was absent from the data.

The provision of social support has consistently been shown to reduce the risk of postnatal depression, providing a ‘buffering effect’ that helps women to cope with postnatal stressors and build confidence and self-esteem in their new role [106–111]. However, there is currently a lack of research into perinatal interventions that specifically target loneliness. This study helps pave the way for further exploration of social and psychological interventions that target loneliness in the perinatal period with a further aim to understand their impact on the risk of perinatal depression. This might include the adaptation for perinatal depression of existing successful interventions for loneliness and depression in the general population – for example, those that improve meaningful social connections [89] or attempt cognitive modification to improve subjective isolation [25].

## Conclusions

This meta-synthesis of 27 qualitative studies derived eight meta-themes describing experiences of loneliness among women with perinatal depression. Whilst few of the included studies specifically asked women about loneliness, the frequency with which it came up in women’s accounts demonstrates the centrality of loneliness in relation to perinatal depression. Women described how feelings of loneliness often arose due to self-isolation in response to the perceived stigma associated with their depression and the fear of being judged as a ‘bad mother’. Women reported their internal critical self-comparisons and resulting feelings of failure led to a sense of inauthenticity and fraudulence when interacting with their peers. A sense of disconnection with their family, babies and with their pre-pregnancy selves created a complicated dual sense of emotional and social loneliness. Women emphasised the importance of regular, trusted support and validation from healthcare professionals, peer support from women with lived experience of perinatal depression as well as non-judgemental emotional and practical support from families. Women from disadvantaged or marginalised communities experienced a double burden with pre-existing inequalities and social isolation interacting with and worsening their experience of perinatal depression and loneliness. The results provide a solid foundation for further theories about the role of loneliness in perinatal depression. They also provide evidence on which the development of future psychological and social interventions could be based, to address the stigma faced by women experiencing perinatal depression and to offer personalised and culturally appropriate support that might reduce the risk of both loneliness and depression.

## Supplementary Information

**Additional file 1: Search strategy and Table 1.** Free text and MeSH terms used for search in Ovid MEDLINE®.

**Additional file 2: Table 2.** Extended table with full details and characteristics of included studies.

**Additional file 3: Table 3.** Extended table of themes with comprehensive supporting primary data.

## Data Availability

The datasets used and/or analysed during the current study available from the corresponding author on reasonable request.

## Abbreviations

CASP: Critical Appraisal Skills Programme
EPDS: Edinburgh Postnatal Depression Screen
GP: General Practice
MBU: Mother and Baby Unit
NEET: Not in education, employment or training
PAG: Patient Advisory Group
PND: Postnatal depression
PPD: Postpartum depression.
