# Supplementary material for: ‘Just snap out of it’ – the experience of loneliness in women with perinatal depression: a Meta-synthesis of qualitative studies
Source: BMC Psychiatry. 2023 Feb 28;23:110. doi: 10.1186/s12888-023-04532-2 (PMC9970854; doi:10.1186/s12888-023-04532-2)
Supplement: Supplementary file 1 — Additional file 1: Search strategy and Table 1. Free text and MeSH terms used for search in Ovid MEDLINE®. [file 12888_2023_4532_MOESM1_ESM.docx]

**Appendix 1:**

**Search strategies and lists of search terms including modifications used for search in Ovid MEDLINE ®**

The initial search protocol included search terms relating to all perinatal mental health disorders to scope the available literature. However, as per the specified inclusion and exclusion criteria, only studies relating to women with perinatal depression were included in the final analysis.

The search terms were modified slightly for specific search engines with database-specific filters used where appropriate; however overall semantics were not altered. An example of all the search terms including free text and MeSH terms used for search in Ovid MEDLINE is below in Table 5. Asterisks were used to broaden the search to include variations of a term. Both MeSH and free-text terms were used for the search, using the PICO framework, and were connected by Boolean operators “OR” and “AND”.

The following search terms were used combining free text and MeSH terms for each concept using the Boolean operator OR. Subsequently each concept was combined using Boolean operator AND (i.e. “1 AND 2 AND 3 AND 4”):

**Table 1: Free text and MeSH terms used for search in Ovid MEDLINE ®**

| Concept (combined using AND) | Free text (combined using OR) | MeSH (combined using OR) |
| --- | --- | --- |
| 1. Perinatal population | perinatal or peri-natal or postpartum or post-partum or antenatal or ante-natal or prenatal or pre-natal or postnatal or post-natal or puerper* or pregnan* or gestation or peripartum or peri-partum or parturition or childbirth or child-birth or childbearing or child-bearing or matern* or mother or baby or babies or infant | exp maternal health services/ or maternal health services.mp.  or exp Perinatal Care/ or perinatal.mp. or exp Pregnancy/ or pregnancy.mp. or exp Peripartum Period/ or peripartum.mp. or exp Prenatal Care/ or prenatal.mp. or exp Postpartum Period/ or post- partum.mp. or exp infant/ or infant.mp. or infant, newborn.mp. or exp infant, newborn/ |
| 1. Mental health disorder | mental* health* OR mental disorder* OR mental* ill* OR mood disorder* OR psychiatr* OR depress* OR anxi* OR bipolar disorder OR bipolar OR post traumatic stress OR post?traumatic stress OR schizo* OR psychosis OR eating disorder* OR anorexi* OR bulimi* OR personality disorder* OR obsessive compulsive OR bab* blue* | exp Puerperal Disorders/ OR puerperal disorders.mp. OR mental health.mp. OR exp Mental Health/ OR psychiatry.mp. OR exp Psychiatry/ OR mental disorder.mp. OR exp Mental Disorders/ OR exp Depression/ OR exp Suicidal Ideation/ OR exp Cognition Disorders/ OR exp Self-Injurious Behavior/ OR exp Borderline Personality Disorder/ OR exp Mood Disorders/ OR exp Anxiety Disorders/ OR exp Depression, Postpartum/ OR postpartum depression.mp. |
| 1. Loneliness | lonel* or perceived social isolation or social isolat* or emotional isolat* or social network* or social support or social contact or social relation* or social capita or alienat* or social interact* or perceived social support or subjective social support or social connect* | loneliness.mp. or exp Loneliness/ or perceived social support.mp. or exp Social Support/ or exp Social Isolation/ or social isolation.mp. |
| 1. Qualitative study | Qualitative* or lived experience or interview* or focus group* or IPA or interpretive* or grounded theory or narrative* or Discourse* or Thematic* or Content Analysis or Ethnograph* or Phenomenolog* or Hermeneutic or semistructured* or semi-structured or unstructured* or guided interview* or guided discussion* or group discussion or transcribe or open-ended or mixed method or mixed-method or framework approach | qualitative research.mp. or exp qualitative research/ or grounded theory.mp. or exp grounded theory |
