# Supplementary material for: ‘Just snap out of it’ – the experience of loneliness in women with perinatal depression: a Meta-synthesis of qualitative studies
Source: BMC Psychiatry. 2023 Feb 28;23:110. doi: 10.1186/s12888-023-04532-2 (PMC9970854; doi:10.1186/s12888-023-04532-2)
Supplement: Supplementary file 2 — Additional file 2: Table 2. Extended table with full details and characteristics of included studies. [file 12888_2023_4532_MOESM2_ESM.docx]

**Appendix 2:**

**Table 2: Extended table with full details and characteristics of included studies**

| **Author & ref** | **Year** | **Study aim** | **Participants** | | | | **Study setting** | | **Data collection & analysis** | | **Quality assessment (score out of 10)** |
| --- | --- | --- | --- | --- | --- | --- | --- | --- | --- | --- | --- |
|  |  |  | **Sample size** | **Participant characteristics** | **Diagnosis** | | **Country** | **Clinical setting recruited** | **Data collection method** | **Data analysis method** |  |
| Beck(27) | 1992 | To describe postpartum depression as it is experienced in every day life and to answer the research question: what is the essential structure of the lived experience of postpartum depression? | 7 | Age range 22 - 38 yrs. 3 with college degrees & 4 with high school diplomas. | Postpartum depression | Self report | USA | Postpartum depression support group | One-to-one interviews | Colaizzi's phenomenological 6-step data analysis method | 6 |
| Blanchard(110) | 2009 | ﻿To capture the phenomenological essence of the experience of depressive symptoms during preg- nancy from the couple’s perspective. | 7 | Mean age = 24 yrs (range 18-29). 2 married & 5 cohabiting. All Caucasian. EPDS scores 10 - 20 | Antenatal depresssion | EPDS>=10 | USA | Family health centre - primary care & counselling | Semi-structured interviews | Colaizzi's phenomenological data analysis method | 9 |
| Boath(53) | 2013 | ﻿To elicit and explore the experiences of teenage mothers with postpartum depression focussing on their experiences of being a teenage mother; support needs and the potential for support and education to be delivered by healthcare workers, or peers. | 15 | Mean age = 18.8 yrs (range 17-19). 10 single, 1 married, 4 cohabiting. EPDS scores 13-28 (mean 18). 3 further education students, 12 NEET. | Postpartum depression | EPDS>12 | England | Health visitors in the community | Semi-structured interviews | Thematic framework analysis | 9 |
| Edhborg(54)** | 2005 | The aim of this study was to explore and describe how Swedish women with signs of postpartum depression two months postpartum experience the first months with their child. | 22 | Mean age = 32.3 (range 20 - 42 years). 13 married & 9 cohabiting.. 17 Swedish & 5 non-Swedish. Median EPDS 12.5 | Postpartum depression | EPDS>=10 | Sweden | Maternity ward | Unstructured interviews | Grounded theory | 9 |
| Evans(72) | 2012 | ﻿To explore the perceived value and types of social supports that characterize the discussions women who participate in PPD online support groups | 512 online postings (number of contributors unknown) | Anonymous users of an online postpartum depression support group | Postpartum depression | Self report | Based in Canada but participants could be international | Online postpartum depression support group | Unit of analysis was individual online messages | Directed content analysis | 7 |
| Gardner(63) | 2014 | ﻿To explore the lived experience of postnatal depression (PND) in West African mothers living in the United Kingdom (UK) | 6 | Age range 22-36 years. 3 Nigerian women & 3 Ghanaian women. 4 married & 2 single. | Postpartum depression | EPDS>=10 | England | NHS commissioned parenting groups. | Semi-structured interviews | Interpretative Phenomenological Analysis | 8 |
| Hanley(69) | 2006 | To examine the experiences of Welsh mothers diagnosed with postnatal depression and to question whether postnatal depression is socially determined. | 10 | Age range 17-33 years. 6 married; all except 1 co-habiting with partner. | Postnatal depression | EPDS>=12 & diagnosed by GP | Wales | General Practice/ primary care. | Semi-structured interviews | Content analysis using Colaizzi's method | 6 |
| Highet(28) | 2014 | To gain insight into women’s lived experience of postnatal depression and anxiety, the factors that contribute to these symptoms and the context in which they develop. | 28 (24 face to face & 4 telephone) | 14 under 35 years old & 10 over 35 years. 26 married or in relationship & 2 single parents. 18 in employment & 10 not in employment | Postnatal depression | Self report | Australia | Website of 3^rd^ sector organisation | Face to face and telephone interviews | Grounded theory | 8 |
| Jackson(58) | 2020 | To explore women’s experiences of living in rural northern England and receiving care from extended family during periods of perinatal mental illness. | 21 | 1 aged 16-24 years, 10 aged 25 - 34 years & 10 aged 35 to 44 years. 20 married or co-habiting with partner, 1 in a relationship but not co-habiting. | Antenatal or postnatal depression. | Self report | England | Flyers, health visitors, midwives, support groups and local press. | Semi-structured interviews (18 face-to-face & 3 telephone) | Thematic analysis | 10 |
| Keefe(111) | 2016 | To learn how the faith, church participation, and spiritual practices of new African American and Latina mothers with histories of postpartum depression (PPD) helped them cope with PPD. | 30 | Age range 18 - 44 years. 19 African Americans & 11 Latinas. Average EPDS score 18.75. Between 1 and 11 children living with them. | Postpartum depression | Self report | USA | Flyers in an urban health centre serving low-income residents. | Semi-structured face-to-face interviews | Constant comparative analysis | 4 |
| Keefe(68) | 2019 | To examine how neighborhood and relationship violence, intermittently involved fathers, and isolation contribute to mothers’ perinatal depression. | 30 | Mean age = 28.6 yrs (range 18 - 44). 19 African American & 11 Latinas. Between 1 and 11 children living with them. | Postpartum depression | Self report | USA | Flyers in an urban health centre serving low-income residents | Semi-structured face-to-face interviews | Constant comparative analysis | 5 |
| Letourneau(60) | 2007 | ﻿To assess the support needs, support resources, barriers to support, and preferences for support intervention for women with postpartum depression. | individual interviews = 41; group interviews = 11 | Mean age = 31.27 yrs. 88% married. 81% white. 29% maternity leave, 12% full time work, 20% homemaker. 27% completed university. 85% English language. | Postpartum depression | Self report | Canada | Newspapers and community health care clinics | Semi-structured interviews & focus group interviews | Thematic content analysis | 9 |
| Mauthner(57) | 1995 | To explore the role that relationships between mothers plays in the processes of developing depression and how they later overcome it | 18 | Age range 20 - 39 years. Range of educational backgrounds. 11 full time mothers, 3 part time work, 4 full time work. 16 married & 2 co-habiting. 100% White | Postpartum depression | Self report | England | Details not available | Semi-structured interviews | Voice centred relational method | 5 |
| Mauthner (67)** | 1998 | To explore and understand postnatal depression from the mothers' points of view by prioritizing their own accounts of their experiences. | 18 | Age range 20 - 39 years. Range of educational, socioeconomic & occupational backgrounds. All co-habiting with father of chid. 100% White. | Postpartum depression | Self report | England | Local and national organisations, support groups, health clinics & 'network sampling' | Semi-structured in-depth interviews | Voice centred relational method | 6 |
| Montgomery(61)** | 2009 | To describe women's understanding of their partners' or husbands' involvement in the midst of PPD. | 27 | Age range early 20s to mid 30s. All prescribed medication for depression. All co-habiting with partner. Parenting 1 to 4 children. | Perinatal depression | Self report | Canada | Peer support groups, community mental health agency, public health agency & family play centres | Unstructured interviews | Narrative analysis | 7 |
| Morrow(55) | 2008 | To explore in the Canadian context the experiences of three groups of first-generation Punjabi-speaking, Cantonese-speaking, and Mandarin-speaking immigrant women with depression after childbirth | 18 | Age range 27-49 yrs. 3 Punjabi-speaking, 8 Cantonese- speaking & 7 Mandarin-speaking immigrant women. Range of 1-3 children. | Postnatal depression | Self-report | Canada | General and family practitioners, community-based organisations, | Semi-structured interviews | Ethnographic narrative approach | 8 |
| Nahas(56) | 1999 | ﻿To explore the lived experiences of postpartum depression among the Middle Eastern women living in Sydney, Australia. | 45 | Mean age 28.6yrs (range 19-38). 18 Lebanese, 14 Egyptian, 13 Palestinian. 22 1^st^ time mothers, 12 2^nd^ time mothers, 11 3rd to 5th time mothers. None in paid employment | Postpartum depression | Self report | Australia | Arabic community centres | Unstructured interviews | Phenomenological study | 9 |
| O'Mahony(74) | 2012 | ﻿How do immigrant and refugee women utilize available health care services and social support networks to cope with PPD and its related problems? What services or strategies could address PPD care and treatment among immigrant and refugee women? | 30 | 8 refugee women and 22 immigrant women. Diverse ethnocultural backgrounds. 25 married or cohabiting, 4 separated & 1 single. Majority homemakers | Postpartum depression | EPDS>=10 | Canada | Health care providers & organisations that provide mental health services | Semi-structured interviews | Critical ethnographic method | 10 |
| Raymond (65) | 2009 | ﻿To explore the feelings of depression during pregnancy of a local sample of women living in an area of socio- economic deprivation, and to identify the support mechanisms that they report as personally or potentially helpful for antenatal depression | 9 | Mean age 29 yrs (range 23 - 40). Between 1-3 children. Mixture of living with partner or no contact 6 White, 2 Black, 1 Mixed Asian/British. Living in area of high deprivation | Antenatal depression | Self report | England | GPs, nurseries, health visitors | Semi-structured interviews | Thematic analysis | 9 |
| Recto(71) | 2020 | ﻿To examine the types of social support received by pregnant and parenting Mexican-American adolescent mothers and their perceptions of how it can influence perinatal mental health. | 20 | Mean age 17.15 yrs (range 15-19). Identify as Mexican American. 60% living with parents or father. 80% first pregnancy | Perinatal depression | Self report | USA | Parenting classes - school nurses and social workers helped recruit | Interviews | Deductive content analysis | 7 |
| Roseth(64) | 2011 | To explore the meaning of depression through mother's experience | 4 | Median age 30 (range 24-40 years). All cohabiting or married. | Postpartum depression | EPDS>=13 or clinical diagnosis | Norway | Local healthcare clinic nurses or psych outpatient dept | Interviews | Descriptive-phenomenological method | 6 |
| Scrandis (73) | 2005 | ﻿To generate a theory of how women experiencing postpartum depressive symptoms mobilize social supports. | 10 | Mean age 32.5 (range 26-39). 9 had 4+years of college education; 9 were White, 1 was African American. All married | Postpartum depression | Self report | Unknown | Community sites - churches, postpartum support groups, home visiting programs | Semi-structured interviews | Grounded theory | 5 |
| Shafiei(70) | 2015 | ﻿to investigate immigrant Afghan women's emotional well-being and experiences of postnatal depression after childbirth and their use of health services. | 39 telephone interviews; 10 face-to-face interviews | Immigrant Afghan women. Telephone interview - 11 < 25 years & 7 > 35 years; 97% married; 38% first baby; 62% completed year 12; all Muslim; 41% good English; 51% 5 years or less in Austraia. | Postnatal depression | EPDS or self report | Australia | Antenatal clinics or postnatal wards | Semi-structured telephone interviews then face-to-face in depth interviews | Thematic analysis | 9 |
| Tang(59) | 2016 | ﻿How does Chinese culture influence the relationship between social support and PPD? | 38 | Mean age 31.5 yrs (range 24-38). All married (on average 5.4 years), 78.9% working | Postpartum depression | Self report | China | Convenience sample from community | Semi-structured interviews online | ﻿Grounded theory approach and constant comparison method | 6 |
| Taylor(18) | 2021 | To explore the ways in which loneliness and isolation feature in women’s narratives of perinatal depression, or how this may vary for different women. | 14 | Age ranges 1 over 40 and 3 under 25. 8 White British, 1 Mixed Race, 2 Black Caribbean, 2 Black African, 1 Arab. 7 living with partner. 6 university degree or higher, 2 no qualifications. | Perinatal depression | Self report | England | NHS health providers from primary care to acute and secondary mental health care | Semi-structured interviews | Social constructionist theory. Thematic analysis. | 10 |
| Templeton (75) | 2003 | ﻿To describe the experiences of women suffering from postnatal depression in black and minority ethnic communities in Wiltshire, UK. | 6 (+additional 14 from focus groups) | Women from Black & Minority Ethnic communities - Bangladeshi, Indian, other Asian & 'other' (2 Portuguese & 1 Mixed Race) | Postnatal depression | EPDS>=12 | England | Local community known to health viitors | Semi-structured interviews + focus groups | Descriptive thematic analysis | 6 |
| Wittkowski(62)** | 2012 | To better understand the experience of postnatal depression in South Asian mothers living in Great Britain by asking (a) what factors led to and maintained how they were feeling and (b) how they defined and experienced what is meant by the term “PND”. | 10 | South Asian origin (5 Asian Indian, 4 Asian Pakistani, 1 Asian Bangladeshi). Mean EPDS score 19.6 (range 16 -23) | Postnatal depression | EPDS>=12 | England | Health visitors and midwives. | Face to face interviews | Constant comparison and grounded theory approach | 8 |

Key: EPDS = Edinburgh Postnatal Depression Scale; **=Papers identified by hand searches; PND= postnatal depression; PPD = postpartum depression; NEET = Not in education, employment or training
