# Supplementary material for: ‘Just snap out of it’ – the experience of loneliness in women with perinatal depression: a Meta-synthesis of qualitative studies
Source: BMC Psychiatry. 2023 Feb 28;23:110. doi: 10.1186/s12888-023-04532-2 (PMC9970854; doi:10.1186/s12888-023-04532-2)
Supplement: Supplementary file 3 — Additional file 3: Table 3. Extended table of themes with comprehensive supporting primary data. [file 12888_2023_4532_MOESM3_ESM.docx]

**Appendix 3:**

**Table 3: Extended table of themes with comprehensive supporting primary data**

| Meta-theme | Sub-theme | Primary data |
| --- | --- | --- |
| 1. Self-isolation, hiding depressive symptoms and loneliness due societal stigma | *1.1 ‘Just snap out of it’ – Societal stigma about perinatal depression and fear of judgement as a ‘bad mother’ makes women feel lonely* | ‘*If a woman shows any signs of being unable to cope, she is considered a “bad” wife … I have to show them [family] that I am coping very well and that I am happy*.‘(56)  ‘…. women felt guilt and like a ‘‘bad mother’’ if they were not immediately happy about the new child but instead wanted personal space and time for themselves.’(54)  ‘It was clear from women’s narratives that many experienced a sense of failure or inadequacy that could prevent them from connecting to others. The mothers described feeling under pressure—from themselves, partners, family members, other mothers, and wider societal narratives—to take on the role of a primary caregiver and to be, as Lottie put it, “a perfect mum”’(18)  ‘*My baby was not able to latch on properly, and I was very anxious. Every time I breastfed my baby, my mother-in-law stood next to us and stared. I felt enormous pressure.*’(59)  ‘Mothers voiced a fear that healthcare professionals could take their children away from them if they revealed that they had postnatal depression and were judged as unable to cope. ‘(53)  ‘Among our participants, the mothers who were single, young, deprived, or from ethnic minority backgrounds (i.e., those who were mothering in what they experienced as more disadvantaged social positions), seemed to experience a particularly acute fear of being judged to be ‘bad mothers’’(18)  ‘The accounts of some women, in particular two women, June and Anaya, who both identiﬁed as black African and Muslim, suggested that the wider stigma around mental health difﬁculties in their culture could also increase isolation. June and Anaya each said that, in their cultures, having depression (whether you were a mother or not) was seen as “*Your fault, whatever, you’ve done a mistake*.”’(18)  ‘Compared to the urban mothers, rural mothers perceived greater stigmatization associated with PPD, perhaps associated with the challenge of preserving anonymity in small communities.’(60) |
|  | *1.2 Self-isolation & hiding depressive symptoms compounds loneliness* | ‘… *I didn’t want to talk with anybody about it, I always had to pretend that I was doing just great … I thought that wasn’t normal, that I was a bad mom who felt that way… (Primipara)*’ (54)  ‘The older mother of three described how she resorted to ‘hiding’ the way she felt: *I was frightened to tell anyone, but things had been getting on top of me. I thought it was just lack of sleep and this heavy cold. I thought that after a good night’s sleep it would get better and I would be able to manage again.* (Elaine*)*’(69)  ‘The participants described a withdrawal from others by hiding their true thoughts and feelings by isolating themselves. One mother described vividly how her strong feelings of shame made her actively conceal her real feeling and thoughts.. Another mother described how she exhausted herself by putting on a happy mask and doing her best to keep up appearances’(64)  ‘There was also a tendency in depressed women to isolate themselves. Women described a strong desire to not have to leave the house but rather be alone and for others to go away: ‘‘*I didn’t want to see anyone – even though I needed support I just wanted everyone to leave me alone’’*(28) |
| 2. Sense of emotional disconnection associated with perinatal depression fuels loneliness | 2.1 ‘*Inauthentic relationships’ with other mothers and feeling misunderstood* | ‘‘*This girl I knew . . . I said “Oh, do you feel like that, do you?” and she’d say “Oh, no, no, no” and I said “Oh-oh, pull yourself together dummy . . . you’re alright.” and then I’d get home and think “She doesn’t feel like that, perhaps it isn’t normal” . . . . That was the one thing that really got to me through it all, that I couldn’t find anyone who felt like I did, and I felt like I was going through it on my own . . . . I couldn’t find anyone who said “Oh yes, I felt like that, don’t worry, you’ll get better” . . . . I felt really isolated and lonely through it*.’ (Pam)’(57)  ‘In these initial contacts, the women described how they “checked out” their feelings with their new female friends and enquired about their experiences of the birth feeding the baby, loving the child, the child’s behaviour, and so on. They felt none of these other mothers echoed their own difficult feelings, and they therefore came to feel isolated within their experiences*:’ I remember going to [a postnatal group] . . . andI.. .thought... “You shouldn’t be here.” I just felt everyone else seemed so happy and so really pleased with their labours, and I remember saying to one girl “How did you findit?“.. . and she [said] . . . ‘found it much easier than I thought” and I burst into tears on the spot. So I didn’t go there again*.’ (Vera)’(57)  ‘Women perceived that other mothers were coping better with motherhood. When new mothers met in social situations formally (e.g., at new mother ’ s groups) or informally (e.g., shopping), there was neither a readiness nor a willingness to discuss PPD.’(60)  ‘…shame connected to anxieties that they were inadequate mothers. There was a sense of loneliness in the mothers’ beliefs that they were alone in their feelings, and ought not admit to them, preventing honest, authentic interactions with others.’(18) |
|  | *2.2 Disconnection from baby* | ‘‘Several women also described difﬁculties bonding with their babies, for example feeling “nothing”, “numb”, “terriﬁed of them” or like their baby “wasn’t a part of me”. For some mothers, a baby’s need to be close to them could feel uncomfortable, even threatening, yet a lack of closeness also resulted in high levels of distress. As Emma explained: “[*My baby] used to have to sleep on my chest and, because I didn’t want him near me, it was really hard having him on my chest. So he used to just lay there and scream in pain and I used to just sit in another part of the room and just cry*.”’(18)  ‘*I just expected to fall in love with him totally, straight away, which I didn’t… I hate myself for that, I felt ever so wicked… I felt really tortured about that…. That I didn’t love him. I know it’s not that unusual…. With people who even haven’t got depression, but I felt evil for not feeling the way I thought I would… I cuddled him, ‘cos I felt I ought to, not because I particularly wanted to…. I was going through the motions…. But my heart wasn’t in it and I always was aware of it, and it just felt like an act, I felt like a fraud.’*(67) |
|  | 2.3 *‘Dislocation’ in sense of self after birth* | ‘At work, some mothers felt they could not share their new experiences of parenthood with colleagues who were not parents themselves. Several mothers felt alienated from colleagues who disapproved of working mothers’(57)  “She began to feel low and associated this with the sudden change in her life after giving birth, describing herself as, “*Terriﬁed of having a day with no plans*”. She struggled to feel connected, integrated and capable at home with her baby, no longer having a routine or role in which she felt competent.’(18)  ‘….feelings of dislocated identity clearly included a strong sense of loneliness and desolation, as the mothers described themselves becoming conﬁned to their homes with their babies, isolated from the wider world, and disconnected from their past lives and social networks. A dislocated self was particularly evident in the narratives of ﬁrst-time mothers who had stopped working or taken maternity leave to have a baby.’(18)  ‘LK*: It would be useful to discuss your problems and what’s going on and how you feel, because nobody seems to ask you that now you have got a baby, it’s ‘how’s the baby?’,it’s never ‘how are you today?’*.’(53)  ‘*Having zero enjoyment for life, it as dark, it was a dark space. . .nothing made me smile, and nothing made me happy, nothing made me enjoy anything’…… ‘It was interfering with our whole life. I’d make arrangements with friends and I would never go*’(28)  “*I just kept thinking about all the things that I couldn’t do anymore and wishing for the life I once had... it’s about like, identity*. ”(18) |
| 3. Mismatch between expected and actual support associated with loneliness | 3.1 *Subjective lack of emotional and practical support from the wider family and community* | ‘Caroline discussed that her mother who lived in the next town had not offered day-to-day companionship or the emotional support she had expected. Subsequently, living in a rural village where she said there were few women with young children and baby clinics run irregularly, meant she spent long periods on her own with her baby. She said she lacked someone to turn to for emotional support, advice and reassurance. ‘*I thought that I might have done some more things with my Mum… I felt she just didn’t really have a lot of time to do things with me, and didn’t really want to… She’d sometimes just bob [pop] in for literally five minutes, then just go again and be like, “Oh, I’m going.” I felt a bit, not hurt,… I felt a little bit disappointed really.*’ (Caroline, postpartum psychosis and postnatal depression, rural village)’(58)  ‘As Latoya, a 29-year-old African American mother of three, summarized, [*W]hen I was pregnant I had that mindset we’re gonna have a precious family, we are gonna stay here and raise them, you’re going to help me. But . . . it’s always like I gotta carry the burden by myself, so that’s pretty stressful . . . everything is pushed on me and I never get a break*.’(68)  ‘My mother works, so I didn’t think it was fair to ask her to mind the children. She has her own life to lead too’(69).  ‘The women at one of the groups said that cultural restrictions meant they could rarely go out on their own or do certain things without being accompanied by another family member (one woman was only allowed out on her own to go to the shops or to attend the support group); these can be contributory factors to feelings of depression.’(75)  ‘Anna left a safe, secure social network back home to ﬁnd a very precarious situation in her new country: *The biggest problem that I faced was arriving late in my pregnancy and didn’t have a specialist. So when it’s time for delivery they said go to the ER . . . I was so stressed the words were gone and was unable to speak. Everything is new . . . you are alone and not with your family and don’t know their system, so this is scary . . .*’(74)  ‘Participants attributed causes of PND as social in origin reporting a lack of support within the community: *I think it is about the stress……. and the [lack of] community……* – Participant 5’(63) |
|  | *3.2 - Lack of support and gender imbalance in partner relationship* | ‘Women spoke about feeling isolated and alone and overwhelmed with anxiety by being the sole caregiver for their infants while their partner was at work.’(60)  ‘‘Women often spoke about their difficulties in verbally articulating their suffering so that their husbands understood their needs. As M3 explained, "*I don't even know how to explain it/it is hard to bring up the words."* They acknowledged that difficulties in expressing themselves clearly contributed to family chaos and strained marital relations.’’(56)  ‘Most mothers spoke of negative influences on their marital relationship due to less time for each other or for their mutual interests, and they did not talk and touch each other as much as earlier.’(54)  ‘While Lottie said her partner “*stopped contact . . . and decided to move on to a new relationship*”, she was left struggling to look after their baby alone. Lottie felt that parenthood had little impact on her partner’s life or social status, whereas for her the impact was profound.’(18)  ‘*The mother is left to do it all...cleaning, ironing, dusting, the man may occasionally make food, but that is seen as a favour. I think that ‘new’ men are very few and far between*.' (Sammy)’ (69)  ‘She explained how she was often alone with her daughter when she was feeling unwell: *I don’t really have a support network, it’s my husband, and that’s it”.... But I was really low ... I felt like I couldn’t look after my daughter properly, which wasn’t the case, because she’s perfectly fine. She was with me most of the day, because my husband worked, he’d leave at half one, and get home just after midnight.* (Jess, preexisting anxiety and depression and post-natal depression, rural town and fringe)’(58) |
| 4. Validation from trusted healthcare professionals | ‘*Because your doctor is someone you have to trust. You can tell them what you’re feeling and they won’t tell anybody. I trust her (health care provider) and I’m completely comfortable with her. With professional help, they don’t criticize you… They know about pregnancy and what you’re going through… She sat down and talked with me about it (depression)… Talking to her helped me feel better*.’(71)  ‘“*They ask me how I’m doing, if I need anything… It’s like they’re really wanting to help… It makes me feel like they really care*.”’(71)  ‘Lauren, for example, described how it was her stay on an MBU [psychiatric mother and baby unit] that ﬁnally enabled her to bond with her baby because she felt cared for herself in a safe place that was “*kind of like family*”.’(18)  ‘RB: *you don’t always want to talk about everything when others are there because you are wondering what they are thinking about me*. TF: *It’s scary sitting in front of other people and talking about your problems, because you don’t know what people are thinking bad against you, so it’s nice to have someone to speak to privately’*(53)  ‘When support from the extended family was insufﬁcient, the most appreciated source of support was from the voluntary sector, in particular the ‘Home-Start’ scheme. All the mothers had been offered the services of this nation-wide voluntary service. Seven mothers accepted the service, which included weekly visits from a volunteer, who was normally herself an experienced mother. The Home-Start support groups addressed the physical and social isolation.’(69) | |
| 5. Peer support from other mothers with perinatal depression | ‘It made me feel so good when they told me they felt that same way … they both had it [PPD]. It was very comforting to listen to them tell me that they understood how I felt and that it was going to get better and it would pass and that I wasn’t a bad mother. That helped.’’(60)  ‘The women in this study stressed the importance of knowing that other mothers also were experiencing depressive symptoms. These connections contributed to improving their self-confidence as a mother. Susan described this process: *‘I had another mom at the playgroup who said she had such a bad day yesterday. She felt like screaming her head off, and I said, "Oh yeah? I had that the other day." I'm not the scum of the earth because I feel this way. I'm normal*.’(73)  ‘Penny, for example, returned to work 4 months after her son’s birth, because she felt inadequate as a mother and incapable of looking after him. Against her will, she worked for a year, feeling extremely isolated during this time. She felt unsupported at work: “*Some of [my work friends] were nasty because I went back to work I don’t know if I’d still call them my friends*,” she said. As a result of working, Penny did not become involved with the “mumsy crowd,” as she called it, and felt she “missed so much” during that year. When she eventually left work, she “really got into the mumsy crowd” and said, “*now, I’m just one of them and it’s great because I’ve got someone to talk to*.” Leaving her job and becoming integrated into a network of other mothers marked the beginning of Penny’s journey out of her yearlong depression.’’(57)  ‘Participant 1 suggested a group where pregnant women could meet socially for informal support. When asked if a health professional should lead this she said: *There should be someone there who could answer questions, maybe get the group going and then just the group could continue to meety, so the women could get to talk freely amongst themselves about issues that are concerning them*. (Participant 1)’(65)  ‘All participants within this study were recruited from mother and baby groups. For those mothers who were feeling particularly isolated, these groups provided not only a source of support and knowledge but also gave women a sense of community, which is embedded within collectivist West African culture: ……… [*when you start going to the group] you know that you are not alone. So many mothers are going through what you are going through. And some are even MORE than yourself…….. [I think] there should be a gathering for mothers……. So you can chat with another mother.…. it does help*. – Participant 1’(63) | |
| 6. Practical and emotional support from family | ‘Health services often did not accommodate women’s children during treatment sessions, and therefore some women relied on informal childcare to enable them to access this formal support*. I’ve been having counselling every week and I’ve come on in leaps and bounds… my Mum and Dad kept these two (children) and it’s so helpful*. (Tina, postnatal depression and post-traumatic stress disorder, rural village)’(58)  ‘*He (father of the baby) was like, “Well, don’t worry about working, just take care of the baby.” And then I didn’t have to worry about that, working or anything. I didn’t have to worry about work-related things… He helped me a lot with the baby because I was basically doing it on my own*.’(71)  ‘Participants who felt most supported revealed that those close to them offered instrumental support without being asked and intervened when they saw that their help was needed rather than waiting for the mother to ask for help.’(60)  ‘*When I’m depressed, my mom just – she basically cries it out with me. And she just tells me, “Don’t worry about it. It’s just one day, just today. It’ll be better tomorrow,” … I know I can always go to my mom and I can always tell her something and she’s not going to judge me.*’(71)  ‘Mothers frequently mentioned their female friends and relatives, in particular their mothers and mothers-in-law and sisters and sisters-in-law. For example, ‘One time I was feeding the baby in the kitchen and I called my mom and said “ Mom I need you to come over with the kids and I have to go. I need to get some help. ” But, just talking to my mom made me feel better.’(60) | |
| 7. Lack of professional support, groups and facilities | ‘Cindy described her interaction with her obstetrician at her 6-week postpartum visit: *She didn't ask me any questions about how I was feeling emotionally at all. I mentioned to her that I thought I was having a problem with depression and she said "Oh we don't bother to ask people about that because when you are depressed, you don't know you're depressed." I thought that was kind of strange.*’(73)  ‘Other participants however, described their experience with their health care provider as lacking warmth and empathy. “*They’ll ask if you’re okay, and they’ll ask if you’re depressed, but the way they ask you, how they speak and their actions, they’re just asking because they have to ask*.”’(71)  ‘Mothers ( n = 26) described being particularly frustrated and even becoming more ill when health professionals minimized their symptoms. *My interview with [professional] helped, but I think it made me spiral downhill further, because she told me that I was just adjusting to my new job as a mom and was feeling isolated, and I should go back to breastfeeding and enjoy the breastfeeding experience!? And didn’t validate that I’d had anxiety … . So I spiraled downhill because the next day, I was feeling bad again*.’(60)  ‘In addition, one woman mentioned health professionals' attitudes towards Muslims as a barrier to seeking help: *When you're a Muslim and you wear [a] scarf, they think and feel like you don't need or you don't deserve to get help the same way as an Australian or someone who is not Muslim … that makes you feel like I don't want to tell her anything about my problems. What for, she doesn't even like me*. (multipara, 30 yrs old, 15 yrs Aust)’(70)  ‘P4: *I got answers from professionals like, there is nothing wrong with you, go back home stop disturbing us, basically you are wasting our time, and they were horrible. It was a Doctor that said that to me, my husband was sat with me that day as well. I don’t know if they would have said that if I was white*.’(62)  ‘*When I tried to ﬁnd a doctor, they asked me where I was from and when I said “from Mexico” I noticed a change from them . . . there might be some racism. I don’t know. I wanted to run. I felt helpless*.’ (74)  ‘Some women also commented that they felt uncomfortable to talk to a male doctor about their emotional well-being: *‘Because my GP is a man, I don't feel very comfortable telling him everything.*’ (multipara, 30 yrs old, 15 yrs Aust)’(70)  ‘P8: *In Pakistan we only saw lady professionals, but here you don’t have a choice, you have to see the men as well otherwise you don’t get to see a doctor. My husband is always at work so he can’t come with me, I feel very uncomfortable*.’(62)  ‘The generally low number of professionals from minority ethnic backgrounds was also noted’(75)  ‘Participant 3 suffered domestic violence during pregnancy, in addition to signiﬁcant depression, which she did not disclose until her baby was 3 months old. She explained: Every time I went to see the midwife, I always had somebody different, and I don’t want to tell 10 people my story. (Participant 3)’(65)  ‘ “‘*There’s nothing really in this area for mothers. I found it very difficult to meet up locally . . . . I would have liked to have known . . . other mothers around, and if there was sort of a central meeting place in this area’*(57)  ‘She explained that her mental health deteriorated over a month when her health visitor had not visited her because she was ‘over stretched’ owing to the large geographical area she had to cover.’(58)  ‘*There is nothing in my face saying — if you are depressed call this number and all the groups, it seemed, were for breastfeeding mothers or abusive (sic) mothers or mothers that didn’t have money.’*(60)  ‘Lynn drifted away from a group because she felt the moms were "a little cliquish" and not very open to new friendships.’(73)  ‘However, one woman spoke negatively of the group she attended because it tended to be dominated by a group of women from the same ethnic background and she did not always feel that she ﬁtted in’(75)  ‘*I joined a group for women with babies. Everybody was saying, “I’m having this depression and this problem.” I was taking it all in, inside me . . . we will talk about that negativity for an hour and that stays in you . . . I want something positive from somebody else, so then I can think that way rather than thinking* [negatively].’(74) | |
| 8. Conflict and separation from partner, family & community | ‘‘Women often spoke about their difficulties in verbally articulating their suffering so that their husbands understood their needs. As M3 explained, "*I don't even know how to explain it/it is hard to bring up the words*." They acknowledged that difficulties in expressing themselves clearly contributed to family chaos and strained marital relations. M9: ... *and he wasn't hearing me/wouldn't listen to what I was saying/and he was walking away from me/and I grabbed it [a toy] and whack/so hard on my head that it split my head open/and blood starts coming down’’*(56)  ‘It was conspicuous how often depression was linked to relationship turmoil, particularly between women and their partners. The two were often closely interwoven in the mothers’ accounts, as illustrated by Nicole, a mother from a black Caribbean British background who had separated from her husband and who described how they: “*was constantly arguing when we was together, so it was hard to differentiate between our kind of bad relationship and the depression*.”(18)  ‘Eleven of the participants reported being physically abused, and 12 reported being psychologically abused by their babies’ fathers. Latoya explained, “*I always was able to talk to the father of my son, but me and him had a domestic situation where he was hitting me and stuff . . . so I don’t really communicate with him*.” ‘(68)  ‘One woman said that she lived on an army base where there was high incidence of postnatal depression (linked to the husbands being away a lot and the family often having to move around on a regular basis), but that the women on the base are very supportive to each other and this particular woman had made some very good friends.’(75)  ‘Anjelica reported, “[*M]y sisters are in New York City, my father was a drunk, my mother was a crackhead, so you can add it up. . . . I keep my distance from them*.”(68)  ‘In their descriptions of their experiences, it was noteworthy that participants viewed PND as occurring mainly in the UK and not often within African contexts. They attributed their distress to isolation and lack of support.’(63)  ‘Most participants expressed that being a newly immigrated mother without the familiar, preexisting support networks could predispose women to PPD. Women felt that they were vulnerable because of the lack of family support after childbirth. For Anna, being connected was the norm back home: *“The families are so big and so supportive. We’re always in touch . . . always gathering around someone.*” Kate maintained that PPD is more commonly found here because ofthe lack of familial support and the stress of being alone. She felt that isolation and solitude are the biggest problems in developed countries.’(74) | |
